# Enabling QTY Server for Designing Water-Soluble α-Helical Transmembrane Proteins

**DOI:** 10.1128/mbio.03604-21

**Published:** 2022-01-18

**Authors:** Fei Tao, Hongzhi Tang, Shuguang Zhang, Mengke Li, Ping Xu

**Affiliations:** a State Key Laboratory of Microbial Metabolism, Joint International Research Laboratory of Metabolic and Developmental Sciences, Shanghai Jiao Tong University, Shanghai, People’s Republic of China; b School of Life Sciences and Biotechnology, Shanghai Jiao Tong University, Shanghai, People’s Republic of China; c Media Lab, Massachusetts Institute of Technology, Cambridge, Massachusetts, USA; Columbia University

**Keywords:** protein engineering, QTY, transmembrane protein, Web server, protein solubilizing

## Abstract

Membrane proteins, particularly those that are α-helical, such as transporters and G-protein-coupled receptors (GPCRs), have significant biological relevance. However, their expression and purification pose difficulties because of their poor water solubilities, which impedes progress in this field. The QTY method, a code-based protein-engineering approach, was recently developed to produce soluble transmembrane proteins. Here, we describe a comprehensive Web server built for QTY design and its relevance for *in silico* analyses. Typically, the simple design model is expected to require only 2 to 4 min of computer time, and the library design model requires 2 to 5 h, depending on the target protein size and the number of transmembrane helices. Detailed protocols for using the server with both the simple design and library design modules are provided. Methods for experiments following the QTY design are also included to facilitate the implementation of this approach. The design pipeline was further evaluated using microbial transmembrane proteins and structural alignment between the designed proteins and their origins by employing AlphaFold2. The results reveal that mutants generated by the developed pipeline were highly identical to their origins in terms of three-dimensional (3D) structures. In summary, the utilization of our Web server and associated protocols will enable QTY-based protein engineering to be implemented in a convenient, fast, accurate, and rational manner. The Protein Solubilizing Server (PSS) is publicly available at http://pss.sjtu.edu.cn.

## INTRODUCTION

Membrane proteins, which make up approximately 20% to 30% of cellular proteins, are known to play vital roles in most organisms (1). However, membrane proteins are heavily underrepresented in protein data banks due to significant difficulties in expressing and purifying these proteins owing to their poor solubilities (2). As of 1 November 2021, there were 183,584 protein structures in the RCSB PDB (http://www.rcsb.org), and there are only 1,336 unique structures of membrane proteins, including approximately 61 G-protein-coupled receptors (GPCRs) (https://gpcrdb.org) and 35 MFS (major facilitator superfamily) transporters (https://blanco.biomol.uci.edu/mpstruc). The α-helical transmembrane (TM) proteins comprise the largest category of membrane proteins. It is estimated that approximately 27% of all human proteins are α-helical membrane proteins (3), among which the GPCRs have generated considerable research interest (4, 5). Scientists have been resolving the bottleneck caused by the insolubilities of these TM proteins. For example, various detergents have been developed for the purification of proteins. Protein engineering by exhaustive trial and error has also been used to produce soluble proteins. However, few of them have been successful. Computationally aided designs have also been thoroughly attempted. For example, researchers have attempted to make bacteriorhodopsin water soluble using its known crystal structure (6). However, these methods, whether structure dependent or computation-intensive, led to poor results.

In our recent study, which used GPCRs as model proteins, a code-based approach, named the QTY method, was successfully developed for making TM proteins water soluble (7). The method was tested by making several water-soluble GPCRs, which include CCR5, CXCR4, CCR10, and CXCR7. The results indicated that all artificial GPCR variants could be water soluble and maintain ligand-binding activities by slightly changing their *K_m_* values. This method may be used to modify other membrane or nonmembrane proteins containing TM helices, including transporters and ion channels.

Although the QTY strategy is straightforward compared to previous methods, performing QTY design manually is still time-consuming and tedious. Library design, particularly, is impractical to execute without computing power. Moreover, QTY-related bioinformatics analyses are highly labor-intensive, as seven different software programs are involved, and the resulting output needs to be integrated. Therefore, PSS (Protein Solubilizing Server), a Web-based server, was developed to facilitate the QTY method by avoiding the tedious analyses involved. The Web-based server was developed to provide a graphical and friendly interface to a broader group of users. Software programs for QTY-related analysis were incorporated into the server to prepare elaborate analysis reports for QTY design. Using PSS for the typical QTY simple design, a standard analysis report can be completed within 4 min and requires only elementary operations such as copying and pasting. We conducted QTY designs for 825 annotated human GPCRs and 884 TM proteins of Escherichia coli K-12 to test the robustness of the QTY method. The design results were also evaluated, taking advantage of AlphaFold2.

## RESULTS

### Simple design module development.

**FIG 1:**
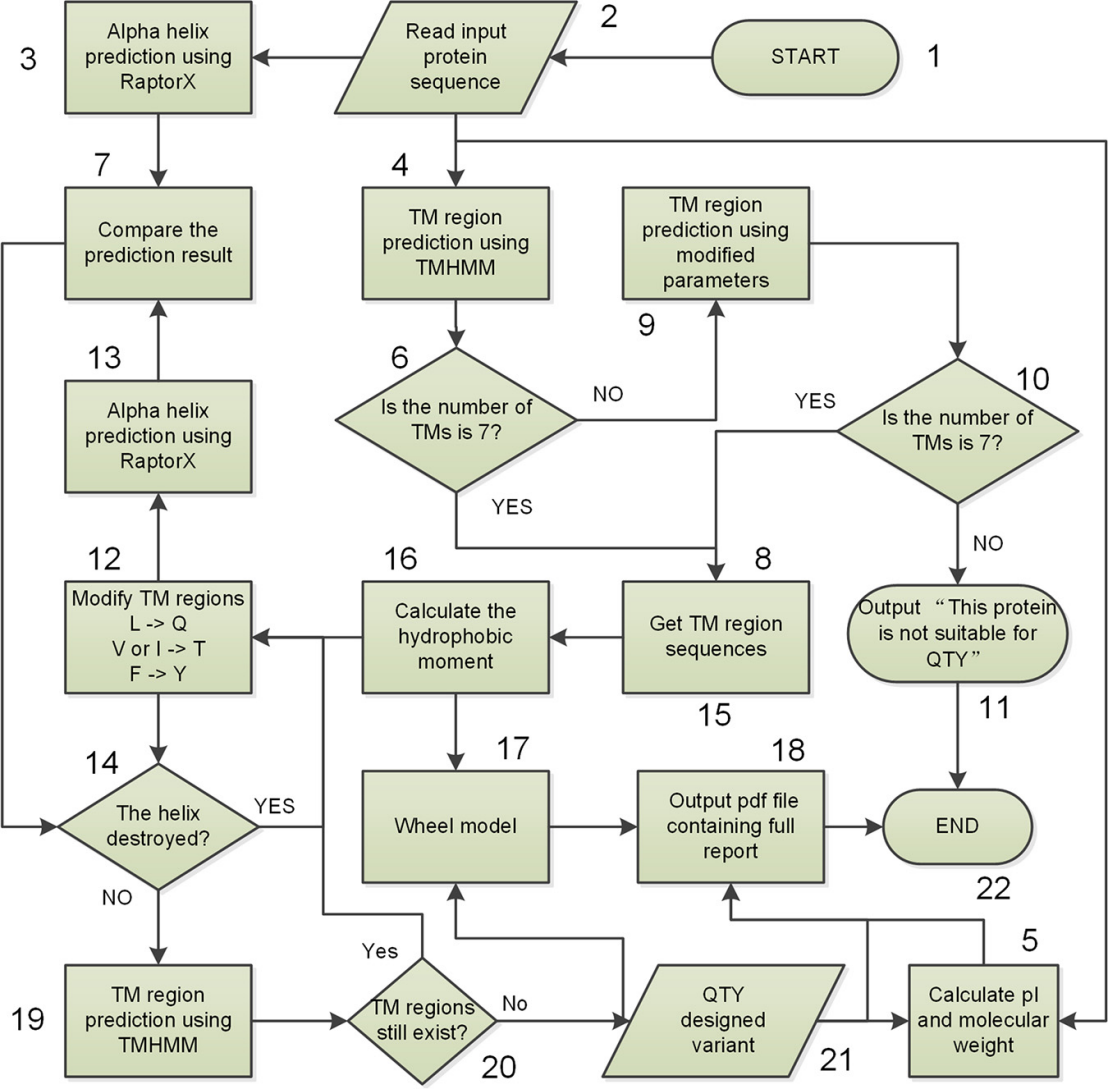
Flowchart for QTY protein design. The workflow for the 7-TM GPCR design was selected as an example to show the computing process of the simple design in cases where only sequences input by the user are available. This is the typical work model of PSS. The process can be divided into 22 steps, including data input and output. All the steps are numbered to facilitate description. “->” means “to.” TMs, transmembrane helices.

To achieve the classical QTY design, a module named simple design was developed. It conducts a direct and complete *in silico* substitution of amino acid residues in a protein with a selected QTY code (Fig. 1). After obtaining the input sequence and TM region information, the server will substitute all changeable residues in the TM regions and output a sequence for the designed protein. The QTY code is selected by default, but it can be changed if a user desires to use the NTY code. The difference between the two codes is that the amino acid N instead of Q is used to substitute amino acid L with the NTY code (7).

After the input, QTY substitution can be completed in seconds because there is no time-consuming calculation step. For simple design, the main use of time is the comparisons that are performed to display details of the design in the output report. There are five comparisons in a standard simple design report (see Text S1, section 3, in the supplemental material). The first one comprises general characteristics, including the calculated molecular weight (MW), isoelectric point (pI), and hydrophobicity (H_Y_). These calculations are made using ProPAS software and the ExPASy server developed previously (8, 9). Comparisons of 15 well-elucidated human GPCRs and their QTY-designed variants are shown in Table 1. All comparison data were directly copied from standard simple design reports.

**TABLE 1:** Selected human GPCRs and their QTY-designed variants

UniProt accession no.^*a*^	Wild type^*b*^			QTY designed^*b*^			R_ACT_^*c*^	R_SC_^*d*^
	MW (kDa)	pI	H_Y_	MW (kDa)	pI	H_Y_		
P41595	54.30	9.22	0.2672	54.76	9.10	−0.7305	0.5484	0.0291
P29274	44.71	8.33	0.4607	45.15	8.29	−0.7072	0.5060	0.1068
P07550	46.46	6.59	0.1700	46.65	6.59	−0.8764	0.4731	0.0630
P51681	40.52	9.20	0.5466	41.05	9.07	−0.9218	0.5576	0.0483
P61073	39.75	8.46	0.3994	40.05	8.40	−0.9113	0.5608	0.0568
P35462	44.22	9.20	0.3190	44.71	9.12	−0.7351	0.4902	0.0750
P41143	40.37	9.20	0.5384	40.75	9.11	−0.6878	0.4629	0.0538
P47871	51.26	9.01	0.0785	51.93	8.90	−0.9388	0.5094	0.0929
P35367	55.78	9.33	−0.0859	56.20	9.24	−0.9675	0.5067	0.0924
P41145	42.65	7.92	0.4816	42.85	7.88	−0.7465	0.5241	0.0500
P41146	40.69	8.73	0.6511	41.14	8.67	−0.4883	0.4720	0.0243
Q9H244	39.44	9.59	0.3512	39.85	9.38	−0.9786	0.5490	0.0263
P21453	42.81	9.58	0.4408	43.38	9.44	−0.9009	0.5506	0.0314
P28222	43.57	8.95	0.3421	43.99	8.88	−0.8378	0.4938	0.0590
Q99835	83.68	8.70	−0.0599	84.17	8.66	−0.5953	0.5102	0.1329

a All 15 selected GPCRs have been well investigated in previous research, and their related structure data can be retrieved from the PDB.

b MW, pI, and H_Y_ indicate molecular weight in kilodaltons, isoelectric point, and hydrophobicity, respectively, which were calculated using ProPAS (8).

c R_ACT_ estimates the changing rate of amino acid sequence in TM regions, calculated by dividing the numbers of changed amino acids by the summarized length of all the TM regions.

d R_SC_ shows the changing rate of secondary structure, which was calculated by dividing the number of positions related to structure changing by the whole length of the protein sequence.

10.1128/mbio.03604-21.1 TEXT S1 Procedures and generated data. Download Text S1, PDF file, 2.1 MB. Copyright © 2022 Tao et al. 2022 Tao et al. https://creativecommons.org/licenses/by/4.0/ This content is distributed under the terms of the Creative Commons Attribution 4.0 International license.

The second comparison is based on TM region prediction, achieved using a standalone version of TMHMM V2.0 software (1, 10). Detailed sequence alignments of a QTY-designed protein and its origin were done using a Perl script, where a Perl SVG module (11) was used to draw the α-helix schematic. Protter interactive protein feature visualization software (12) was also used to demonstrate the differences between the original proteins and the designed ones. This software can also predict TM regions using a serpentine-like map to present the secondary structure pattern. The membrane localization of a protein is also predicted and shown in the output map.

A detailed comparison of each helix was also shown in the report. RaptorX-Property (previously RaptorX-SS8) (13) software, ranked first in secondary structure prediction (14), was incorporated to perform the above-mentioned comparison. The last section of the report revealed sequence alignments of TM regions and alignments of α-helix prediction results. In this section, the helical comparisons were shown using helical wheels of all TM regions. Modified helical wheel drawing software was used to prepare the wheel figure (https://pss.sjtu.edu.cn/cgi-bin/wheel.cgi). All comparisons are integrated and presented in a PDF format report file to be sent to the user’s e-mail address (Text S1, section 3).

### Library design module development.

In the simple design module, all changeable amino acid residues within the TM region(s) are substituted. Therefore, the probability of designing a soluble protein is high. However, there is also a substantial probability that the designed protein may lose its original functions. The rule is that as more amino acid residues are changed, the possibility that the designed protein is soluble will increase, but the chance that the protein is nonfunctional will also increase. Therefore, only the minimum number of amino acid residues that need to be changed to make the TM protein soluble must be changed so that the critical amino acid residues required to maintain protein functions are left unaffected. This underscores the need to find a balance between solubility and protein structure integrity.

**FIG 2:**
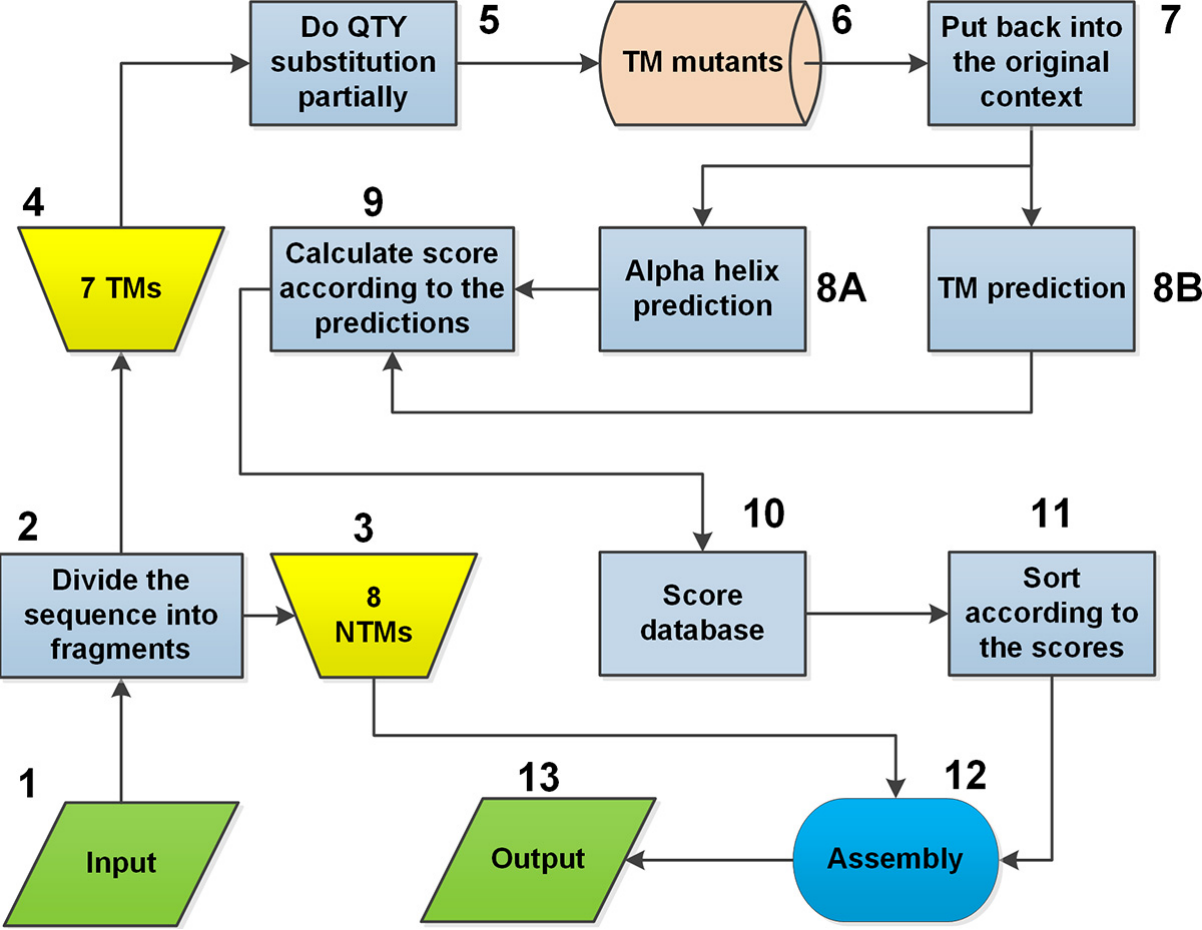
Flowchart for QTY Y2H library design. The workflow of library design is shown using a 7-TM protein as an example. Numbers represent the order of the design steps. There are 13 steps in total, including the input and output steps. NTMs, nontransmembrane regions in a protein; TMs, transmembrane helices.

**FIG 3:**
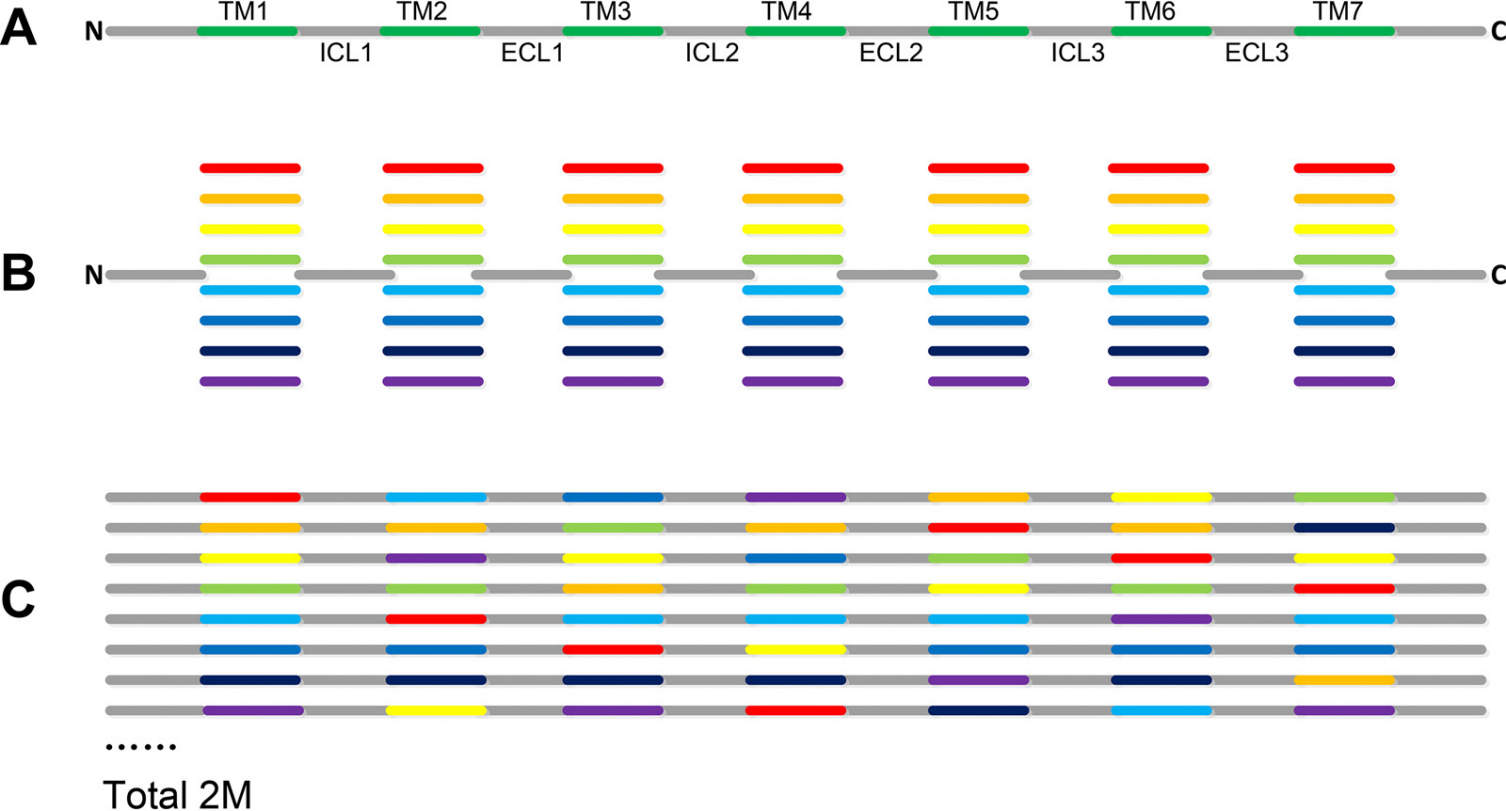
Principle of directed library construction. The design was divided into three steps, A, B, and C. (A) A typical 7-TM protein is divided into 15 fragments, including 7 TMs (TM1 to -7), 3 intracellular loops (ICL1 to -3), 3 extracellular loops (ECL1 to -3), 1 N terminus, and 1 C terminus. (B) Eight variants are designed for each TM. (C) All fragments are assembled, and each variant is used randomly. “Total 2M” indicates the total number of combinations, which is over 2 million (8^7^).

To accomplish the above-mentioned goals, a workflow program (Fig. 2) was developed, based on the domain shuffling principle (Fig. 3), to randomly change TM residues and screen all variants using a high-throughput procedure *in silico*. There are approximately 85 changeable amino acid residues for a typical GPCR. Therefore, theoretically, there may be 2^85^ random variants for a typical GPCR protein, making library construction and screening impractical. Thus, a compromise approach using a partially random method was developed for our server. In this approach, TMHMM TM region prediction software (1) and RaptorX secondary structure prediction software (15) were combined to rule out the most insoluble or nonfunctional variants *in silico*. TMHMM was used to determine if a modified α-helix is water soluble, and RaptorX was used to determine if the modified TM region could still form an α-helical structure. A scoring method was introduced to evaluate the balance. An example of the design of a typical 7-TM GPCR protein is shown in Fig. 3. As shown, there were eight different designed variants for each TM region, and the NTM (nontransmembrane) region was the same as that of the original protein. There were 64 fragments of amino acid sequences in total. Next, the different domains were randomly integrated to form different full-length variants. Calculations indicated approximately 2 million (the 7th power of 8) QTY variants for each 7-TM GPCR library. The *in silico* filtering step in library design is quite time-consuming, typically taking ∼2 h for a single library design of a GPCR containing 7 TM regions.

Considering the time-consuming nature of library design, we deposited 126 previously designed libraries of GPCRs into the server’s database. Some selected proteins screened for their physiological roles in health and disease are presented in Table 2. The complete list of deposited libraries is included in Data Set S2 in the supplemental material. These represent all known human GPCRs that possess protein/peptide ligands. The library designed using these proteins may be screened using the yeast two-hybrid (Y2H) method (16), which is suitable for GPCRs with protein/peptide ligands that have been successfully used in our previous work (7). Using the UniProt accession number as the input and using default parameters, the deposited library designs can be immediately extracted and sent to the user’s e-mail address. Alternatively, the server can run the library design pipeline from scratch and forward an e-mail report to the user upon completion of the job.

**TABLE 2:** Selected 7-TM GPCRs suitable for Y2H library design

Receptor name	UniProt reference accession no. (R)^*a*^	Size (kDa)	Ligand name(s)	Ligand size(s) (kDa)	UniProt reference accession no. (L)^*a*^	Family name
CXCR4	P61073	352	SDF-1α	65	P48061	Chemokine receptors
PAR2	P55085	397	Serine proteases	>100		Proteinase-activated receptors
V3A receptor	P30518	371	Arg-vasopressin	9	P01185	Vasopressin and oxytocin receptors
CCR5	P51681	352	CCL11, -14, -16, -2, -3, -4, -5, -7, -8	74	P51671	Chemokine receptors
ETB receptor	P24530	442	Endothelin-1, -2, -3	21	P05305, P20800, P14138	Endothelin receptors
MC1 receptor	Q01726	317	α-MSH	13	P01189	Melanocortin receptors
ACKR3	P25106	362	CXCL11, SDF-1α	70	O14625, P48061	Chemokine receptors
PTH1 receptor	Q03431	593	PTH, PTHrP	84, 141	P01270, P12272, P12272	Parathyroid hormone receptors
Kisspeptin receptor	Q969F8	398	Kisspeptin-10, -13, -14, -52, -54	10–54	Q15726	Kisspeptin receptor
CT receptor	P30988	508	Calcitonin, amylin	32, 37	P01258, P10997	Calcitonin receptors
CCR2	P41597	374	CCL2	76	P13500	Chemokine receptors
GnRH receptor	P30968	328	GnRH I, GnRH II	10	P01148, O43555	Gonadotrophin-releasing hormone receptors

a The “R” in parentheses stands for receptor, and “L” stands for ligand.

10.1128/mbio.03604-21.3 DATA SET S2 GPCRs with protein or peptide ligands. Download Data Set S2, XLSX file, 0.04 MB. Copyright © 2022 Tao et al. 2022 Tao et al. https://creativecommons.org/licenses/by/4.0/ This content is distributed under the terms of the Creative Commons Attribution 4.0 International license.

There are 2 TXT format files in the standard report for library design (Text S1, section 4). The sequences are separated into multiple lines in one file for ease of printing and reading with a PC/Mac. In the other file, each sequence is presented as a single long line for the convenience of copying and editing using a computer. In our study, single-line files were sent to the company for their service. Notepad++ is strongly recommended for opening the output files. Any other software compatible with TXT file editing may also be used.

### DNA synthesis strategy.

A user of PSS will receive an output of the protein sequence(s). Next, one must design the corresponding DNA sequence(s), which can be used in subsequent protein expression and characterization experiments. In the simple design module, only one sequence is produced by the server. Therefore, a user is required to perform only reverse translation for cloning and expression, taking into consideration translation codon usage bias. This may be achieved with bioinformatics tools such as JCat (17) and OPTIMIZER (18). This work may also be achieved through a commercial service, which is convenient for most users.

For library synthesis, a user can utilize a company specializing in DNA synthesis. In brief, the synthesis process is as follows: each of the 64 fragments of amino acid sequences is reverse translated to the corresponding DNA sequences; next, overlap sequences are added to the ends of each DNA fragment for assembly; and finally, the DNA is synthesized and assembled using PCR. The final PCR product will be a mixture containing approximately 2 million variants (8^7^). This library can then be used in follow-up high-throughput screening such as Y2H screening.

### Convenience evaluation.

With PSS, one can perform designs by simply typing/pasting the sequences of target proteins. A user can also perform designs using only the UniProt accession number of a target protein. There are three ways to input secondary structure data, including the 3-state format SS3 (13) (Text S1, section 2). PSS allows customized designs via input secondary structure data and/or regions that the user wants to modify. With the proper input, PSS can perform the design rapidly. Finally, it produces a detailed report for the design, in which differences between the QTY variant (mutant [MT]) and the wild-type (WT) proteins can be easily checked. A file containing the designed sequence in plain-text format is also provided for gene design and related DNA synthesis.

### QTY design of 825 human GPCRs.

**FIG 4:**
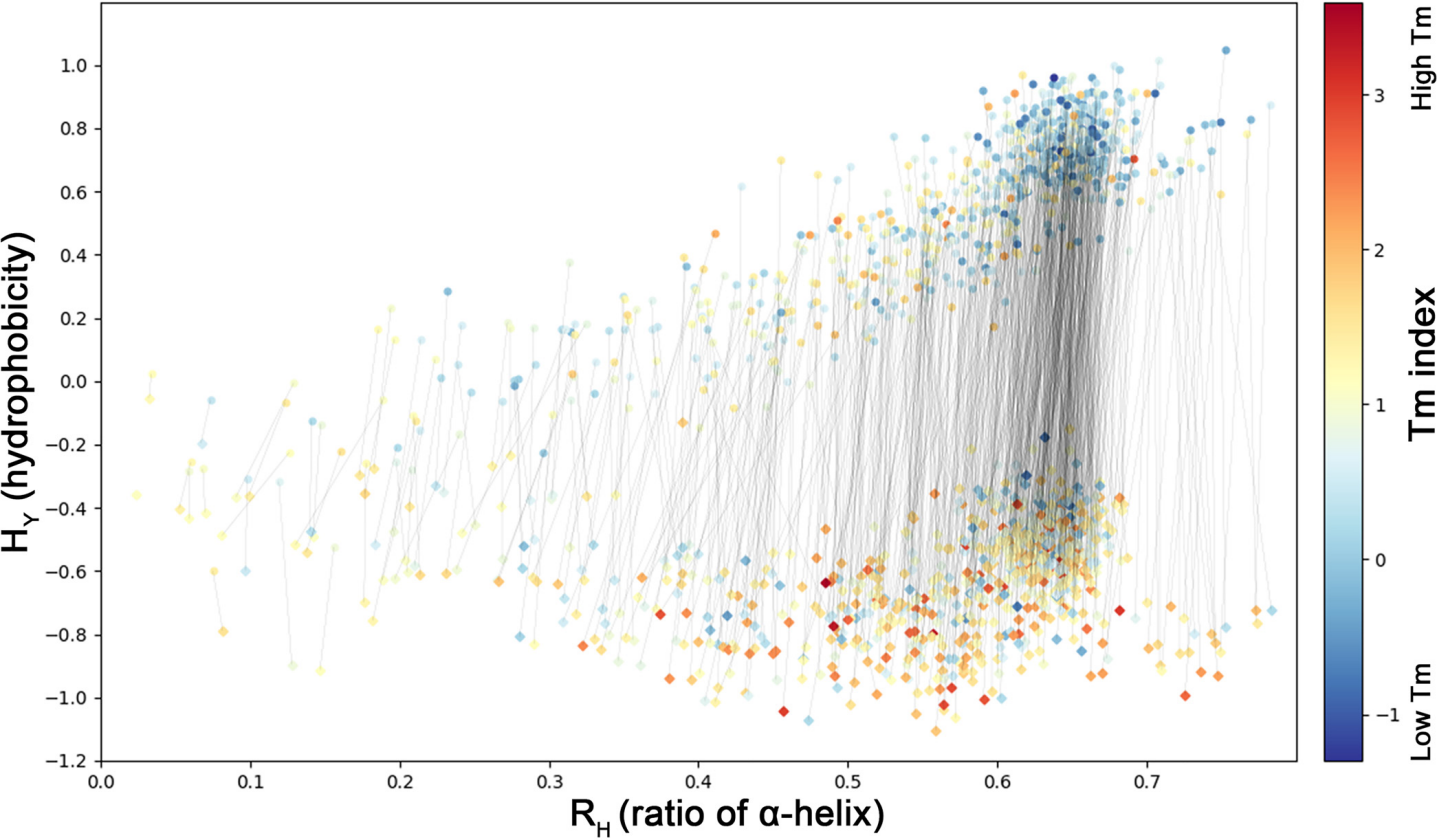
Global R_H_-H_Y_ distribution of 825 human GPCRs and their variants. The R_H_ value indicates the abundance of α-helical regions in a protein. It was calculated by dividing the summarized lengths of all α-helices (including nontransmembrane helices) by the protein lengths. The hydrophobicity (H_Y_) values were calculated using the standalone software ProPAS and then used for evaluating the water solubility of a protein. The *T_m_* index, shown using a color gradient, was calculated using a sequence-based method, which qualitatively represents the stability of a protein. The original GPCRs are denoted by circles, and the QTY-designed variants are denoted by diamonds. The thin black line shows the corresponding relationship between the original protein and its variant. The line slope represents the change rate of the α-helical ratio, which can partially reflect the effect of the QTY design on protein secondary structure.

The development of PSS began in 2013, and the central part was completed in 2016. After 2016, many QTY designs were executed using the server. Experiments aiming to test the design were also performed, including the investigations reported in our previous study (7). We have completed 825 designs on human GPCRs, including related statistical analyses (Data Set S1). A distribution map containing these 825 human GPCRs and their QTY variants is shown in Fig. 4. QTY-designed GPCRs were distinctly separated from their corresponding origins by hydrophobicity (H_Y_), although the α-helix ratios in the whole-protein secondary structure (R_H_) vary from 0.02 to 0.78. Moreover, most thin lines were nearly vertical, suggesting that only minor changes in the secondary structure were produced in the QTY design. Figure 4 also shows that the R_H_ values of most human GPCRs range between 0.5 and 0.7, where the best water solubility improvement was predicted. This suggests that PSS based on the QTY method possesses an excellent capability for solubilizing GPCRs.

10.1128/mbio.03604-21.2 DATA SET S1 Designs of 825 human GPCRs. Download Data Set S1, XLSX file, 0.5 MB. Copyright © 2022 Tao et al. 2022 Tao et al. https://creativecommons.org/licenses/by/4.0/ This content is distributed under the terms of the Creative Commons Attribution 4.0 International license.

The melting temperature (*T_m_*) indexes (19) of QTY-designed proteins were largely higher than their coordinates, indicating that QTY proteins are more stable, which is consistent with our previous observations (7). Designing proteins with enhanced thermostability is a major focus of protein engineering, owing to its theoretical and practical significance (20, 21). Therefore, QTY-based PSS may also provide a general strategy for stabilizing proteins (20).

### QTY design of 884 TM proteins from E. coli K-12.

**FIG 5:**
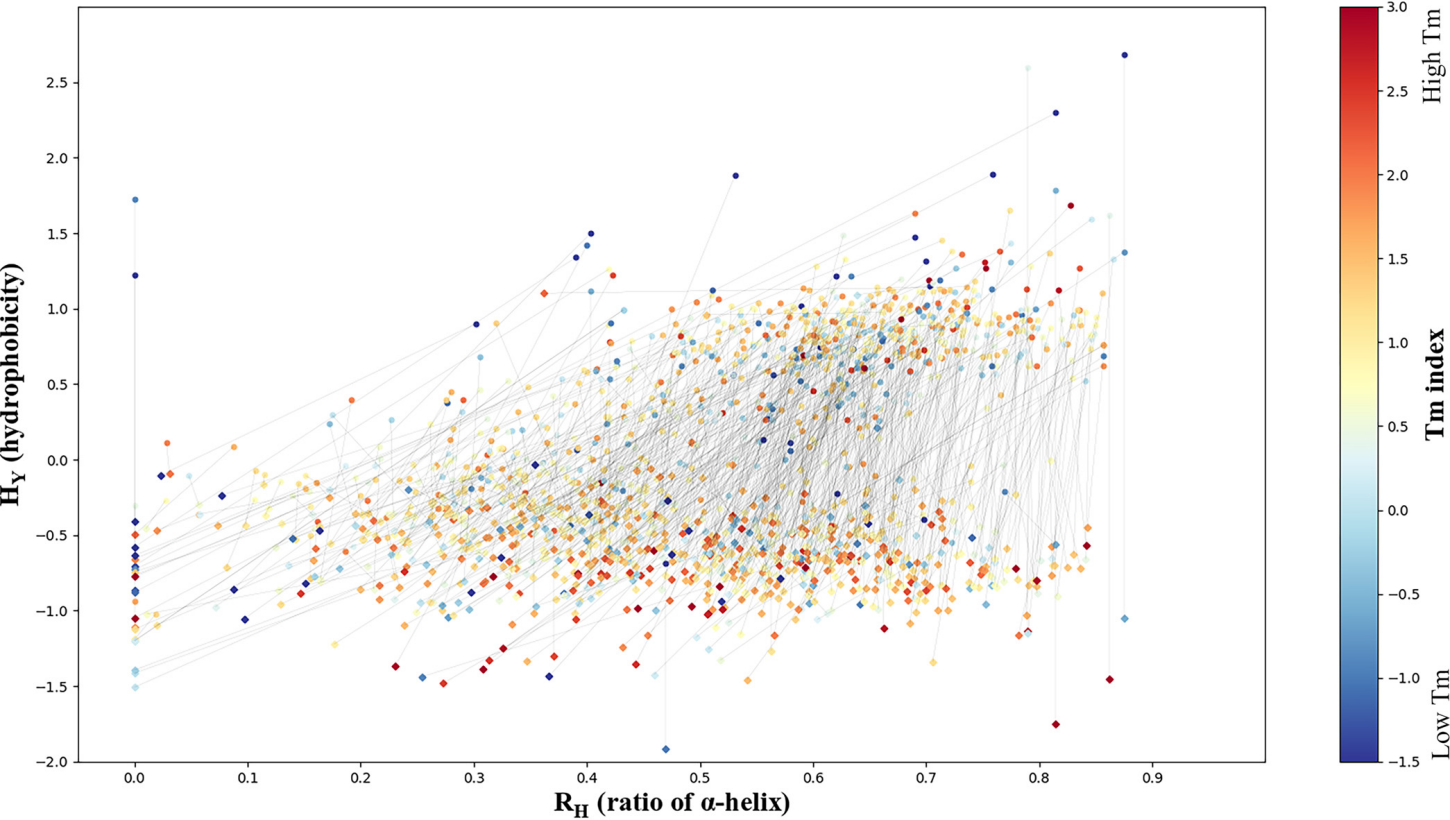
Global R_H_-H_Y_ distribution of 884 E. coli K-12 TM proteins and their variants. The R_H_ value indicates the abundance of α-helical regions in a protein. It was calculated via dividing the summarized lengths of all α-helices (including nontransmembrane helices) by the protein lengths. The hydrophobicity (H_Y_) values were calculated using the standalone software ProPAS and then used for evaluating the water solubility of a protein. The *T_m_* index, shown using a color gradient, was calculated using a sequence-based method, which qualitatively represents the stability of a protein. The original TM proteins are denoted by circles, and the QTY-designed variants are denoted by diamonds. The thin black line shows the corresponding relationship between the original protein and its variant. The line slope represents the change rate of the α-helical ratio, which can partially reflect the effect of the QTY design on protein secondary structure.

There are many types of TM proteins in most organisms. To further test the QTY server, we selected E. coli K-12 as a model organism and performed QTY design for all 884 annotated TM proteins (Data Set S3). A distribution map containing these 884 TM proteins and their QTY variants is shown in Fig. 5. The QTY-designed proteins were separated from their corresponding origins by H_Y_, which was consistent with the results of the QTY design for GPCRs. Like the GPCR design, most of the thin lines were nearly vertical, suggesting that the QTY design for TM proteins of E. coli K-12 may also produce minor effects on the secondary structures. These results suggest that PSS based on the QTY method also possesses a robust capability for solubilizing proteins with different numbers of TM regions. Notably, most of the *T_m_* indexes of QTY-designed proteins were also higher than their coordinates, although the tendency was not as apparent as that of GPCRs. Therefore, it is reasonable to suggest that QTY-based PSS may also provide a strategy for stabilizing proteins with different types of TM passes.

10.1128/mbio.03604-21.4 DATA SET S3 Designs of 884 E. coli K-12 TM proteins. Download Data Set S3, XLSX file, 0.6 MB. Copyright © 2022 Tao et al. 2022 Tao et al. https://creativecommons.org/licenses/by/4.0/ This content is distributed under the terms of the Creative Commons Attribution 4.0 International license.

### Evaluation of QTY design using AlphaFold2.

**FIG 6:**
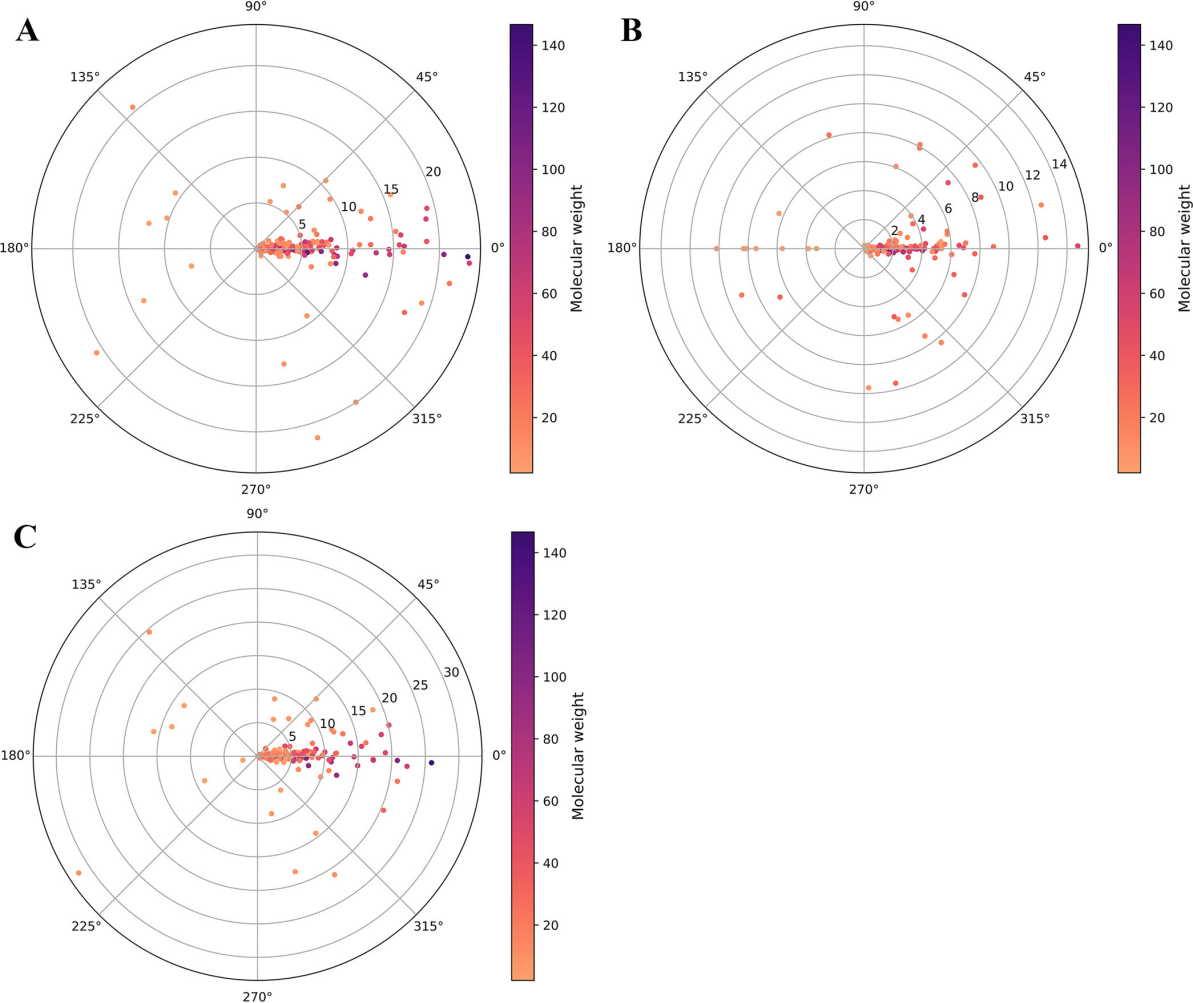
RMSD distribution of the 876 QTY designs for E. coli K-12 TM proteins. Each dot represents a QTY design, with the RMSD value corresponding to the distance from the origin. Above the horizontal line that passes through the origin are proteins with pI values of >7.0, and below are proteins with pI values of <7.0. The angle in the polar coordinate system represents the degree of the secondary structure change. The closer the direction line of a point to the horizontal line to the left, the greater the degree of change in secondary structure. A color gradient represents the molecular weight of each protein. (A) RMSD values of the whole structure. (B) RMSD values of TM regions. (C) RMSD values of NTM regions.

AlphaFold2 has emerged as a new and powerful tool for protein engineering because of its high structure prediction accuracy. To investigate the effects of QTY design on proteins in terms of the three-dimensional (3D) structures, we modeled all 884 QTY variants of the E. coli K-12 TM proteins and compared them with their WT counterparts based on the 3D models. After computation, we obtained 876 successful modeling predictions for the QTY variants, with 8 predictions that failed (22). The structural alignment was done with PyMOL, and the resulting root mean square deviation (RMSD) values are presented in Fig. 6, along with the molecular weights, consistency of secondary structure, and pI values. As shown in Fig. 6, the RMSD values of most QTY designs were <10 (Fig. 6A), which indicated the minor effects of the QTY design on the 3D structures. Notably, the RMSD values of TM regions (Fig. 6B) were even lower, while the RMSD values of NTM regions (Fig. 6C) were nearly the same as those of the entire proteins, suggesting that the QTY-designed proteins were highly consistent with their WT origins in 3D structures. As for the proteins with high RMSD values, it was notable that most of them had low molecular weights. Moreover, the proteins with low consistency of secondary structures also had low molecular weights. These results suggest that QTY design for some small proteins might lead to the collapse of their entire structures.

**FIG 7:**
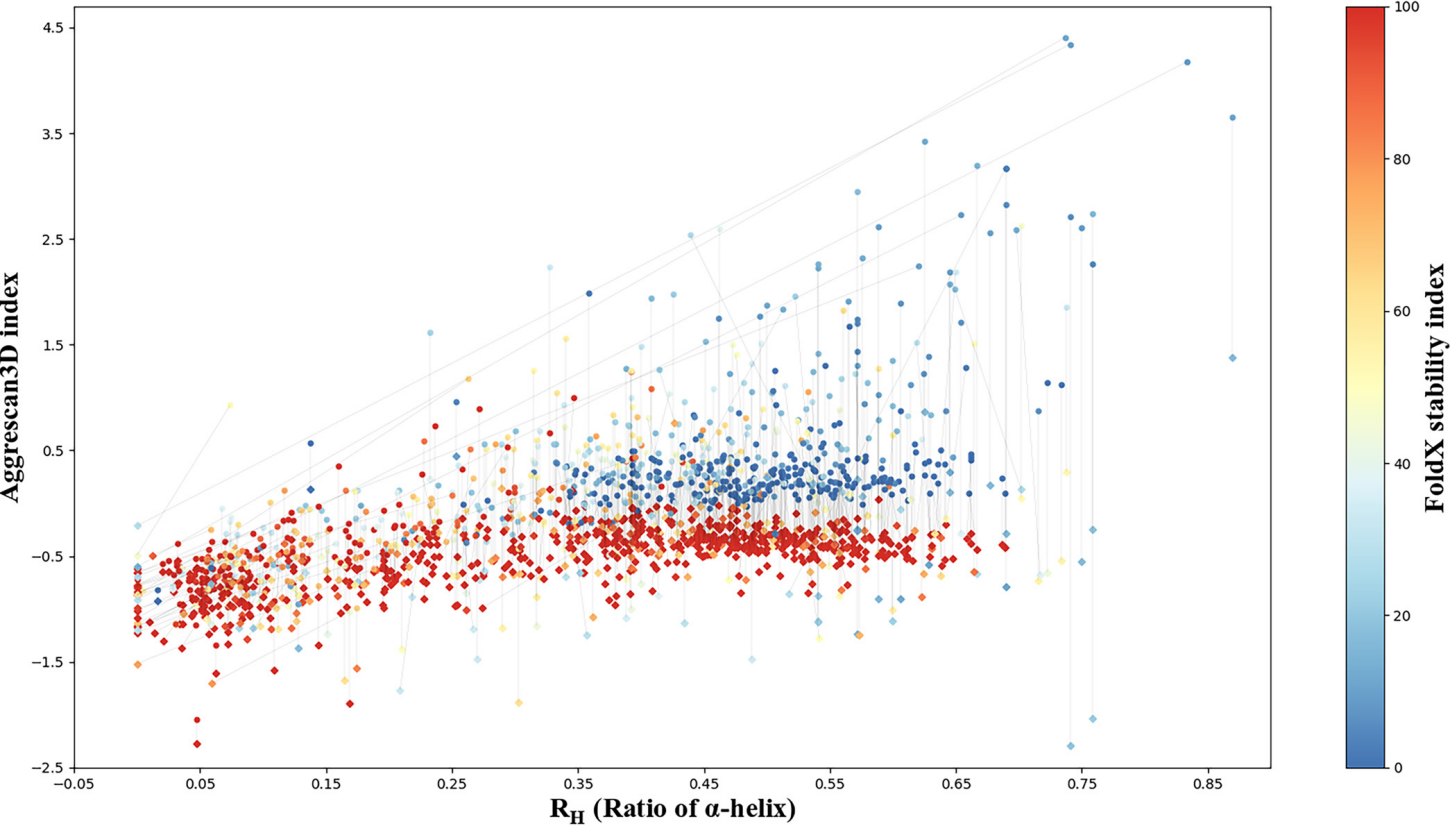
Solubility and stability analysis of 876 E. coli K-12 TM proteins and their variants. The R_H_ value indicates the abundance of α-helical regions in a protein. It was calculated by dividing the summarized lengths of all α-helices (including nontransmembrane helices) by the protein lengths. Solubility is presented by the Aggrescan3D index calculated using a standalone version of Aggrescan3D. Stability is presented by a color gradient according to the FoldX index calculated using FoldX software. The original TM proteins are denoted by circles, and the QTY-designed variants are denoted by diamonds. The thin black line shows the corresponding relationship between the original protein and its variant. The line slope represents the change rate of the α-helical ratio, which can partially reflect the effect of the QTY design on protein secondary structure.

**FIG 8:**
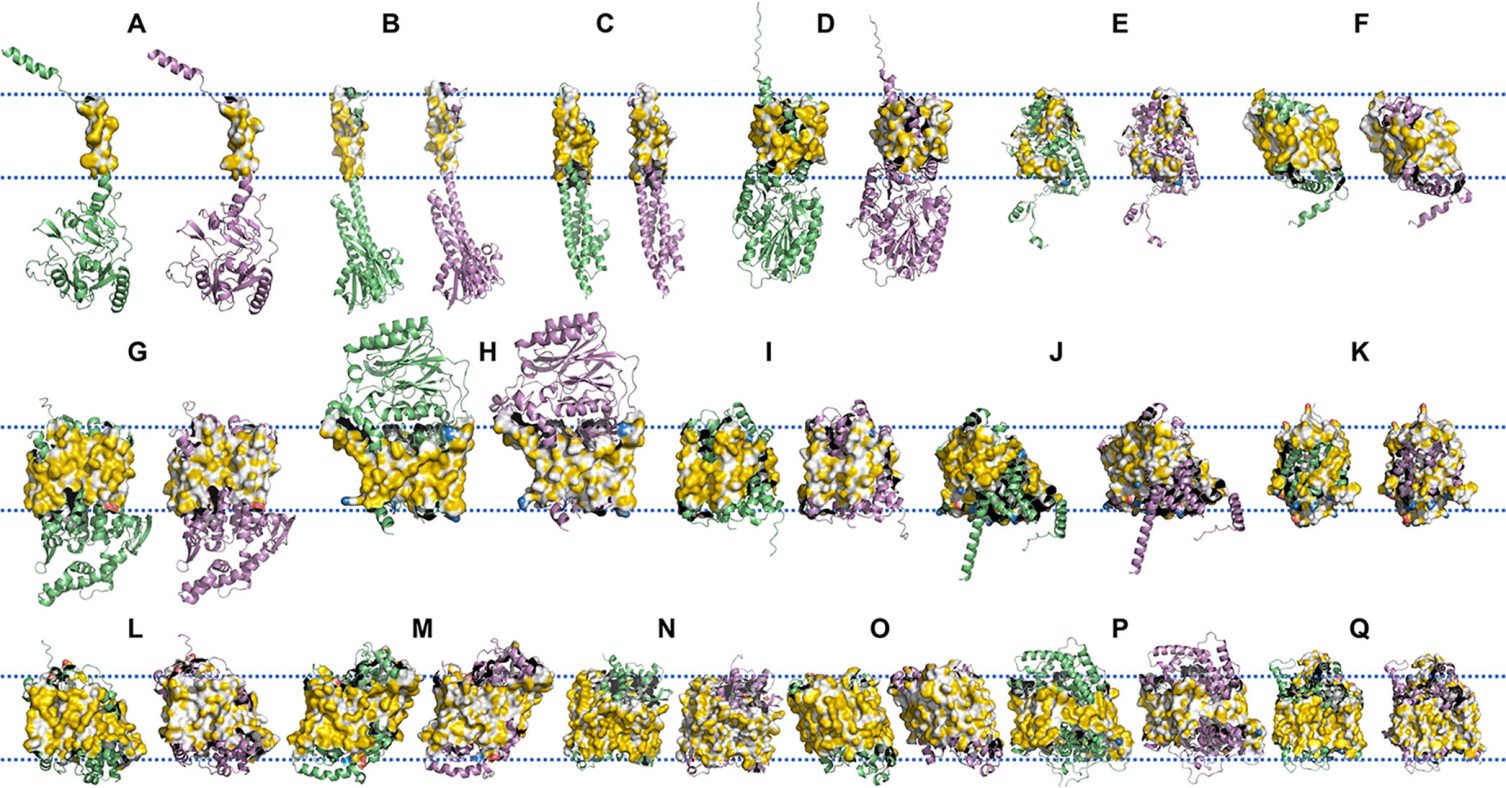
Comparison of selected TM proteins and their QTY variants. The surfaces of TM regions are highlighted according to the YRB highlighting scheme. The double dashed lines represent the plasma membrane. The upper part of the plasma membrane represents the outside of the cell, and the lower part of the plasma membrane represents the inside of the cell. The wild-type proteins are shown in lime green, while the QTY-designed ones are shown in violet. A to Q represent the proteins with the following UniProt accession numbers (names; numbers of TM helices): P0AAJ5 (FdoH; 1 TM helix), P0ABI4 (CorA; 2 TM helices), P0ABU9 (TolQ; 3 TM helices), P0AC26 (NirC; 4 TM helices), P15078 (CstA; 5 TM helices), P23482 (HyfB; 6 TM helices), P23930 (Lnt; 7 TM helices), P25747 (YeiB; 8 TM helices), P26459 (AppC; 9 TM helices), P31553 (CaiT; 10 TM helices), P32705 (ActP; 11 TM helices), P33607 (NuoL; 12 TM helices), P37019 (ClcA; 13 TM helices), P0AFE8 (NuoM; 14 TM helices), P69681 (AmtB; 15 TM helices), P39396 (BtsT; 16 TM helices), and P77416 (HyfD; 18 TM helices), respectively.

To further compare the influences of QTY design on TM proteins, we used Aggrescan3D (23) to calculate protein water solubility and FoldX to calculate protein stability. The corresponding results are shown in Fig. 7. The results showed that the water solubilities of almost all proteins were drastically improved. There was no or only a very small change in the helix ratios of most of the proteins, although the helix ratio of a small fraction of the proteins underwent a significant change, suggesting possible structural collapse. This result was consistent with the above-described results obtained based on protein sequences for GPCRs (Fig. 4) and E. coli K-12 TM proteins (Fig. 5). The calculated stabilities of most proteins were reduced, which is inconsistent with the results observed in Fig. 5. It was reported that FoldX and many similar software programs perform poorly on accuracy to predict the stabilities of membrane proteins (24). It is possible that the poor accuracy in the calculation of wild-type TM proteins resulted in the observed inconsistency. To show the impact of QTY design on proteins more intuitively, we selected 17 proteins and drew their three-dimensional structure diagrams with PyMOL software. As shown in Fig. 8, after the design of QTY, all proteins generally maintained their original structures, while the hydrophobic surface was significantly reduced.

## DISCUSSION

**FIG 9:**
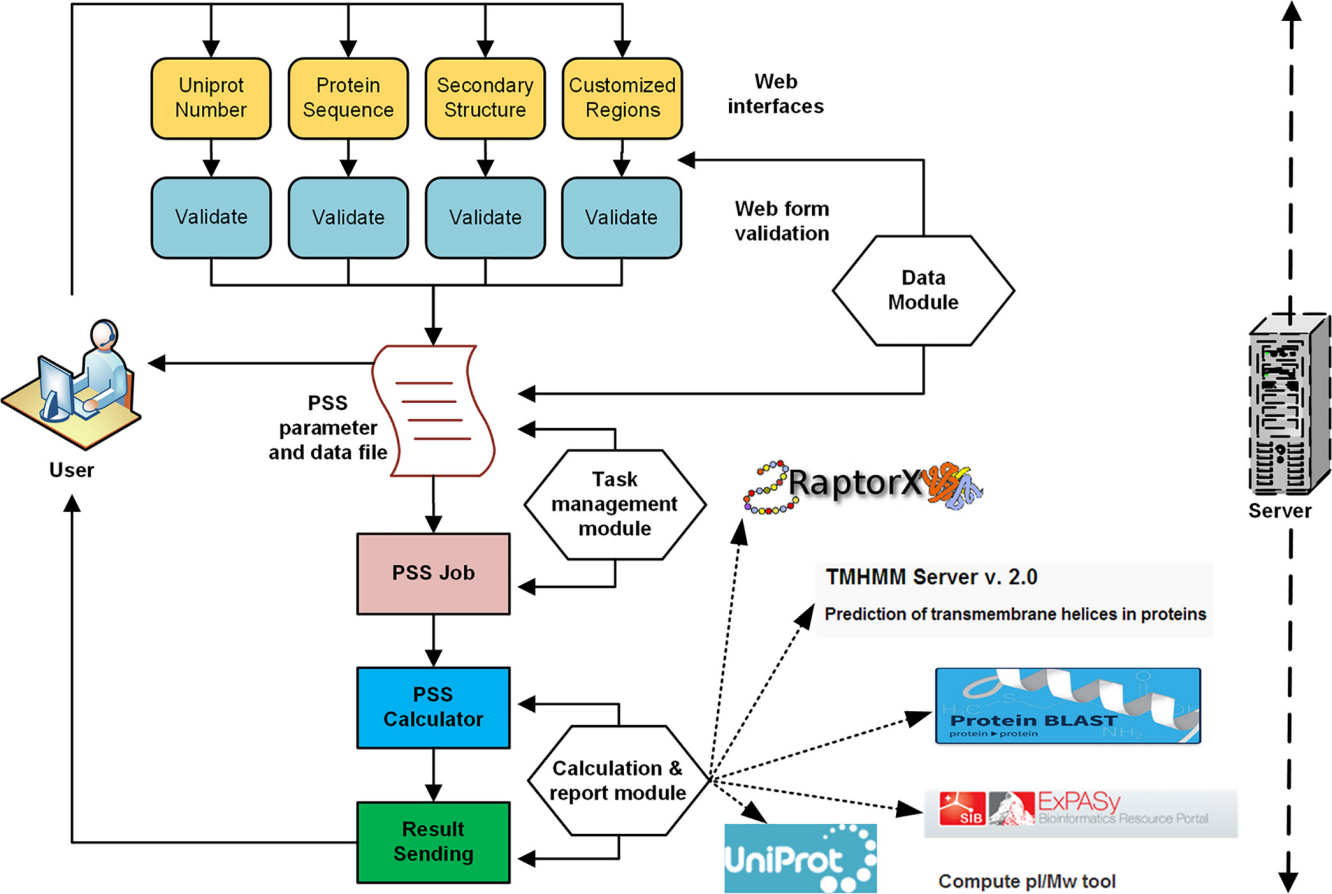
Flowchart of PSS. The Web server consists of 4 parts, namely, the Web interface, the input data managing module (Data Module), the task managing module (Task management module), and the core calculation module (Calculation and report module). In the core calculation module, different software programs were introduced to achieve versatile functions. ExPASy is presented as an example of the software used to predict pI and MW values. ProPAS software and the Protter server are not shown, although PSS also used them. The hydrophobicity values were calculated using ProPAS.

The QTY design was initially conducted with command-line tools written in the Perl programming language, which is executable on various operating systems with a Perl interpreter installed. Because many users prefer a graphic interface, we built the PSS Web server with a broader group of users in mind. It generally comprises two major parts, namely, the simple design module and the library design module, which share the same input requirements (Fig. 9). For the simple design module, users are required to indicate only the region(s) that needs to be changed, and PSS will perform a thorough QTY substitution in the selected region(s). Alternatively, the library design module will perform a partial substitution to maintain the best possible balance between the solubility of the protein and its structural integrity. The library design output can be used to direct library synthesis, followed by yeast two-hybrid (Y2H) screening. The establishment of this server can make it very convenient for researchers to carry out QTY design and obtain professional analysis reports. Researchers can now focus solely on the biology of proteins and ignore tedious bioinformatic operations.

In our previous studies, the effectiveness of the QTY strategy has been demonstrated numerous times for many GPCR proteins (7, 25, 26), and the relevant experimental results were highly consistent with the results calculated by the software (Fig. 4). However, this did not demonstrate the general applicability of the QTY strategy to other types of proteins. Here, we used the model organism E. coli K-12 as an example, and QTY design was carried out on all the annotated TM proteins, including more than 800 proteins with numbers of TM regions ranging from 1 to 18. Next, we used AlphaFold2 to predict the structures of the mutant proteins, based upon which we evaluated the impact of QTY design. The results showed that QTY design has only a small effect on protein structure (Fig. 6). The results also clearly showed that the QTY strategy could significantly improve the water solubilities of most TM proteins (Fig. 7). This calculation was very similar to the results for GPCRs, which indicates the general applicability of the QTY strategy to diverse TM proteins.

Notably, PSS was designed for modifying proteins containing α-helices. Thus, besides GPCRs, any protein with a hydrophobic α-helical secondary structure should be suitable for PSS. There are many such proteins, such as adiponectin receptors, claudin, and tetraspanin. Enzymes with hydrophobic α-helices, such as the cellulase CelDZ1 (27), β-carotene 15,15′-dioxygenase (28), and endo-β-*N*-acetylglucosaminidase (29), are also within the scope of PSS. Although the examples listed above are all TM proteins, the applicability of PSS goes beyond TM proteins, as α-helices are also abundant in nonmembrane proteins. Theoretically, water-insoluble proteins containing hydrophobic α-helical structures might also be solubilized using PSS. For this, a user needs only to manually input secondary structure data according to the instructions described in the procedure section (see Text S1 in the supplemental material).

The server was developed based on theories concerning protein α-helical secondary structure and, therefore, may be used for solubilizing only proteins with hydrophobic α-helical structures. Other types of proteins, such as those with β-barrels, are not suitable for PSS. Another limitation was the computing capability, where only a single sequence per submission was allowed. In addition, each sequence could not contain more than 8,000 amino acids. The library design is applicable only for proteins whose variants can be screened using high-throughput methods such as the Y2H method. Currently, the parameters of library design have been optimized only with GPCRs as the target proteins, and the library capacity is set at 2 million.

## MATERIALS AND METHODS

### Prediction of protein secondary structures.

THMMH 2.0 software was used for predicting the transmembrane helices in a protein and determining the subcellular localization of a protein. Its standalone version was used and run on Ubuntu 16.04 LTS. It was run using the default parameters. To predict the secondary structures of a protein, RaptorX software was downloaded and run on Ubuntu 16.04 LTS (13).

### Calculation of protein properties.

The standalone software ProPAS was used for the prediction of the protein features pI, MW, and hydrophobicity (8). The *T_m_* value was calculated using Tm Predictor localized software with the default *T_m_* reference matrix (19).

### Web server configuration.

The software workflow runs on a Linux server (Ubuntu 16.04 LTS). The main packages used for implementation are TMHMM 2.0, ProPAS, RaptorX-Property, NCBI BLAST 2.6.0+, and Perl 5.22.1 together with Bioperl modules. The responsive user interface is implemented using HTML 5 and JavaScript. Only a computer with an Internet connection and a modern browser (e.g., the latest versions of Chrome, Safari, or Firefox) is required for using PSS. A valid e-mail address is needed for receiving the design results. Adobe Reader and Notepad++ may be used to view the designs. PSS may be accessed at http://pss.sjtu.edu.cn. A detailed procedure that can be followed step-by-step is also provided (see Text S1, section 1, in the supplemental material).

### Input data preparation.

**FIG 10:**
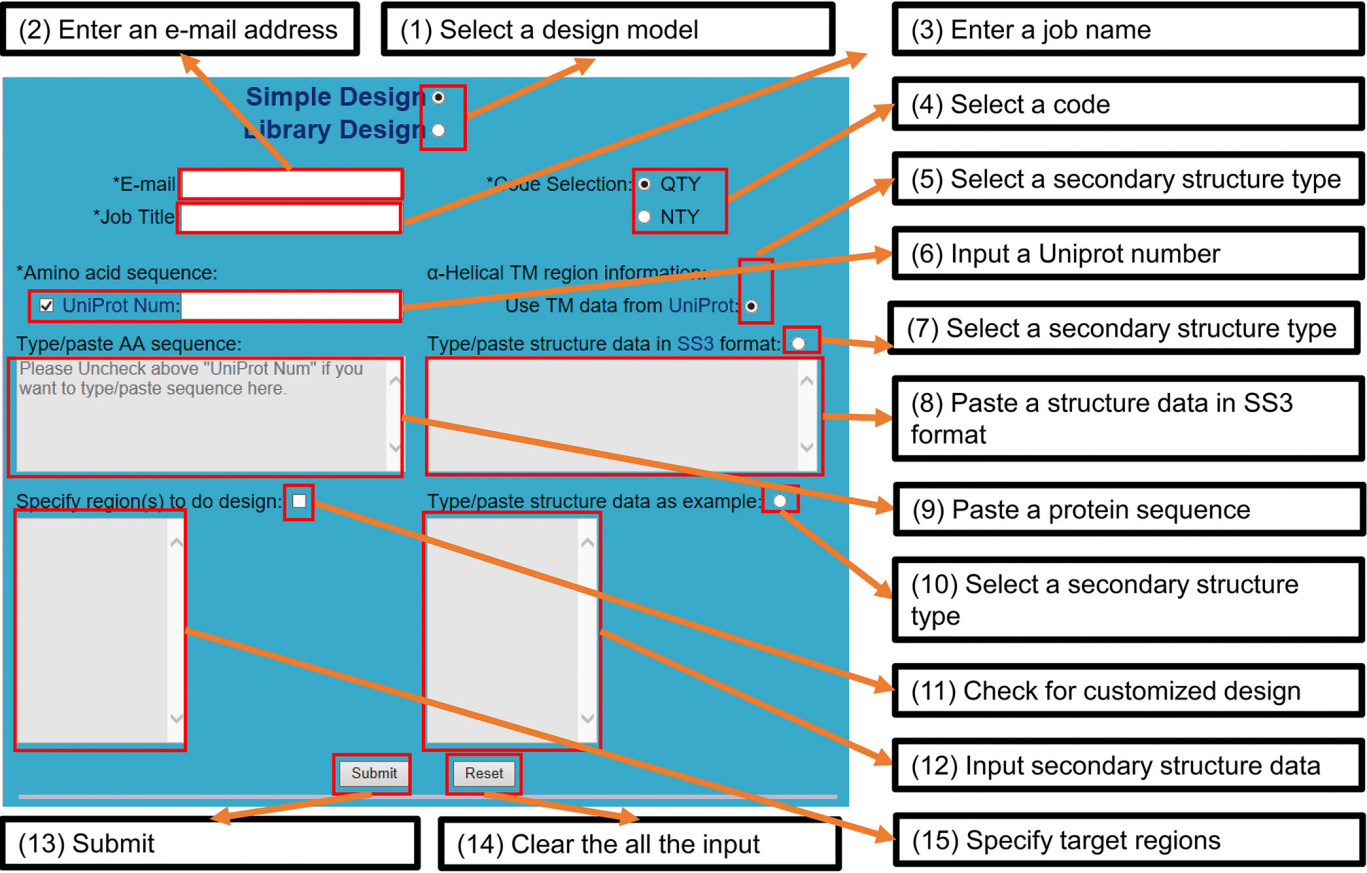
Screenshot of the PSS design page. The major parts of the Web interface are shown. It includes six sections, which contain a total of 15 elements, such as text fields and checkboxes. The asterisks are used to label required items. UniProt is the name of the protein database (https://www.uniprot.org). The “/” between “Type” and “paste” means “or.” The text fields in gray are disabled by default. AA, amino acid.

Two types of information are needed for QTY design. These are the protein sequence and its corresponding TM region information (Fig. 10). By default, the user input must include a UniProt accession number, the unique identifier of a protein record in the UniProt database. Next, the server will retrieve that protein sequence from the UniProt database (https://www.uniprot.org) and use it for QTY substitutions. If a single UniProt accession number represents multiple sequences, only the canonical sequence will be used for the design. Alternatively, a user can also input a protein sequence by typing/pasting that protein sequence into the provided text field (Text S1, section 2).

With regard to TM region information, the design becomes more complicated. Generally, it is difficult to predict the type of data accurately. Therefore, entering TM region information in different ways is allowed. If the UniProt database is used as the data source, the TM region information from the UniProt database will be automatically selected for QTY design (Fig. 10). Alternatively, there are two other exclusively manual ways, which are (i) pasting an SS3-format string and (ii) manually indicating the start and end positions of all TM regions (Text S1, section 2). For typing/pasting a sequence as the input, there are three ways of obtaining the TM region information. Apart from the two manual ways stated above, the server may also obtain TM information by performing TM region prediction using TMHMM V2.0, developed by Anders Krogh and rated as an efficient way to predict TM helices when no external TM region information is available (1, 10, 30).

Considering that users may require a partial modification of a protein at some point, PSS allows the manual selection of specific TM regions to perform a QTY design. For this, a user is required only to input each selected protein fragment’s start and end positions (Fig. 10). In such a situation, the server will perform QTY substitutions only in the TM region(s) within the indicated fragment(s).

### Protein expression method.

Theoretically, any strategy or host for protein expression may be used for expressing the designed proteins. Therefore, users are encouraged to try different methods in performing protein expression experiments. Here, we provide only some advice for users based on our previous experiments. For the expression of QTY-designed human GPCRs, we have used E. coli and insect cells as hosts. Insect cells appear to be highly suitable for expression, while using E. coli always has difficulties related to inclusion body issues. Other host systems, such as yeasts, may also be suitable because the Y2H method always functions well for designed proteins, indicating that yeast expression may be suitable.

### Structure prediction using AlphaFold2.

Protein structure prediction is performed using ParaFold (31) (https://github.com/Zuricho/ParallelFold), a modified version of the newly published AlphaFold2 (22). Shanghai Jiao Tong University developed the software by separating the CPU and GPU parts, with AlphaFold’s MSA construction and use of templates running in parallel on a CPU and training and inference on a GPU. It is considered to be fast without losing accuracy. All the structure prediction jobs were run on computers in the Center for High Performance Computing of Shanghai Jiao Tong University.

### Calculation of solubility, stability, and RMSD based on 3D structure.

We used the standalone version of Aggrescan3D for evaluating the solubility of proteins based on the 3D structure model (23, 32). The stability of proteins was predicted using FoldX (33, 34), which was run on a computer with Ubuntu 18.04 LTS. A Python script was programmed to calculate the RMSD values in batch with PyMOL 2.4.1.

### Protein surface drawing.

We used a script developed by Hagemans et al. to highlight the hydrophobicity and charge on protein surfaces with the YRB scheme (35). Structure alignment was first performed, and the TM regions were then selected for showing the surfaces using the script.

### Evaluation process of QTY design.

When evaluating the QTY design, we used two strategies: one is sequence-based calculations, and the other is structure-based calculations. In the sequence-based strategy, we calculated the property parameters of WT and QTY-designed TM proteins based only on the amino acid sequences. ProPAS software was applied to calculate the hydrophilicity, molecular weight, and isoelectric point. Tm Predictor software was used to calculate the thermal stability of TM proteins. RaptorX software was used for calculating the secondary structure. Next, comparisons were made based on these data. In the structure-based strategy, we first used AlphaFold 2 to generate the 3D structure models for all the WT proteins and their QTY variants. Next, PyMOL was used to compare the TM regions, NTM regions, and the entire protein and to calculate the RMSD values. We used Aggrescan3D to calculate protein water solubility and FoldX to calculate protein stability. Next, various comparisons were made based on these data.
