# Supplementary material for: Enabling QTY Server for Designing Water-Soluble α-Helical Transmembrane Proteins
Source: mBio. 2022 Jan 18;13(1):e03604-21. doi: 10.1128/mbio.03604-21 (PMC8764525; doi:10.1128/mbio.03604-21)
Supplement: TEXT S1 [file mbio.03604-21-t0001.pdf]

SUPPLEMENTARY DATA

PSS: An enabling QTY server for designing water-soluble α-helical transmembrane proteins

Fei Tao[1,2*], Hongzhi Tang[1,2], Shuguang Zhang[2] & Ping Xu[1*]

[1] State Key Laboratory of Microbial Metabolism, Joint International Research Laboratory of Metabolic & Developmental Sciences, and School of Life Sciences & Biotechnology, Shanghai Jiao Tong University, Shanghai, 200240, People's Republic of China.
[2] Center for Bits and Atoms, Massachusetts Institute of Technology, Cambridge, 02139, MA, USA.

* To whom correspondence should be addressed. Tel: +86-21-34206647; Fax: +86-21-34206723; Email: taofei@sjtu.edu.cn. Correspondence may also be addressed to P.X. (pingxu@sjtu.edu.cn)

Section 1:

PROCEDURE

Preparation of required information ● TIMING ~5 min

1 | This step varies depending on a user's situation in real time. In general, 3 types of information are needed:

(A) Amino acid sequence of target protein

Protein sequences to be submitted may be downloaded or copied from any source, such as NCBI. The sequence may be in FASTA format or in string format. Any notation characters embedded in the sequence will be removed automatically by the server. For convenience, the UniProt number (protein record ID) may be used as input. In case the UniProt number is input, the server will retrieve the UniProt database and acquire the canonical sequence. A UniProt number can be obtained by performing a simple search on the UniProt website (www.uniprot.org).

(B) α-Helical TM region information

This section is optional because we have integrated TM region prediction software and TM region retrieving programs in PSS. However, accurate TM region information is critical for an effective QTY design. If a user obtains accurate TM region information from crystal structure data or from another method, we recommend that the data be supplied. PSS accepts 2 types of TM region information data, SS3 format and start-end position format. The SS3 format string can be obtained from many sources such as RaptorX prediction. This format uses 3 letters to indicate the secondary structure of each position, where "H" stands for helix, "C" for coli, and "E" for unknown structure. The start-end position format simply provides the start and end positions of each TM fragments (SD1 section 2).

▲CRITICAL STEP For both formats provided above, all regions labeled as 'helix' or 'H' will be recognized as TM helices and will be exposed to QTY substitution. The α-helix is also known to exist in non-TM proteins. Therefore, in data containing secondary structure of a protein with non-TM α-helices, the section 'Specify region(s) to do design' must be used to accurately indicating TM regions in order to avoid changing the non-TM helices. The secondary structure data may also be edited by only labeling the TM helices with 'helix' or 'H'.

(C) Desired mutant region(s)

This section is also optional, as it is only meant for partial modification of a protein without affecting some of the α-helices. An input box in the webpage was designed to enable start-end position pairs to be entered in order to indicate the regions requiring QTY modification. Thus, PSS will only change α-helices in the indicated regions and leave the other parts as they were in wild type.

Submitting a job ● TIMING 5 min

2 | Go to the PSS homepage at http://pss.sjtu.edu.cn/.

3 | Select 'Design' from the menu at the left of the page.

4 | Select between 'Simple Design' and 'Library Design'.

▲CRITICAL STEP By default, the 'Simple Design' is selected. This is the classical QTY design and can be completed in minutes. The 'Library Design' is only applicable for proteins that can be screened via high-throughput methods.

5 | Supply a job name for identification of different submissions, and an e-mail address in order to receive notifications and design results when the design has been completed.

▲CRITICAL STEP PSS does not require a user to register before submitting a job. Therefore, it is important to provide a correct e-mail address. Otherwise, the results will not be received. The job name is used for identifying each job. It must be ensured that different job names are used for different submissions. Otherwise, only the result of the last submission will be received.

6 | In the 'Code Selection' section, the user need to select a code. By default, the QTY code is selected. An alternative code, NTY, which is considered less effective in making the protein water-soluble but more effective in retaining the native protein structure, is also provided for the design.

7 | The 'Amino acid sequence' contains 2 text fields. The upper field is used to enter the UniProt number which can be used to obtain the corresponding sequence from the UniProt database. The lower text field, which is disabled by default, is for typing/pasting a sequence. The checkbox to the left of 'UniProt number', must be unchecked before typing/pasting a sequence in the text field.

8 | To input TM region information (the secondary structure data), 3 ways are provided. Once the UniProt number is provided, PSS will retrieve TM information from the UniProt database by default. The radio button to the right of ‘Structure data in SS3 format’ can be clicked to activate the text field for typing/pasting SS3 format string. By clicking the radio button after ‘Type/paste structure data as example’ TM data of the start-end positions may be entered.

9 | The section ‘Specify region(s) to do design’ is initially disabled. Usually it can be ignored. In case partial modification of the TM regions are wanted, the checkbox must be selected to activate the text field to enter the start and end positions of the desired fragment(s).

10 | Press the ‘Submit’ button to queue the job on PSS. A successful submission will generate a popup notification stating, ‘Your job has been successfully submitted! The result will be sent to your email address shortly’.

▲CRITICAL STEP Upon submission, the data entered in the form will be validated, and the user will be notified of any errors that need to be corrected in a popup message box. It must be noted that only a limited number of pending jobs are allowed for one user (as identified by their IP address used at submission) in order to maintain sufficient server capacity to serve all users. Specifically, each user may not have more than 20 Simple Design jobs and 5 Library Design pending jobs pending at any point in time. There is no API interface for PSS, and program-aided submission is not allowed.

Job availability ● TIMING 2 min – 5 h

11 | Currently, no job monitoring interface is provided. Therefore, submission of a job is followed by a waiting period. The design result will be sent to the provided e-mail address upon completion of the job. Typically, it takes 2 – 4 min for the Simple Design, while it takes 2 – 5 h for the Library Design. The accurate time cost depends on protein size and number of TM regions. This time cost does not include queuing time.

12 | Frequently check the provided email box until an email titled ‘QTY design for XXXX’ is received. The ‘XXXX’ stands for the job name entered during submission.

Viewing design results ● TIMING 10 – 15 min

13 | This step differs depending on whether a Simple Design (A) or Library Design (B) job is

being submitted:

(A) Viewing 'Simple Design' results

(i) The e-mail contains 2 attachments. Firstly download them to a local file folder.

(ii) The file with a '.txt' extension contains the sequence of the designed protein. It may be easily used in the subsequent gene design and synthesis.

(iii) The file in PDF format consists mainly of detailed comparisons between the QTY designed protein and the original protein. It contains 6 sections, where the first one contains basic information related to the job and the design. The second part is a table showing basic comparisons of the general characteristics of the 2 proteins. The third part comprises a figure containing TM region predictions for both the original protein and the designed variant. Whether the design will make the protein water-soluble may be easily checked. Part 4 is a comparison that indicates the difference between the two sequences at amino acid residue level. Part5 is the comparison of Protter predictions that indicates protein structure changes using a serpentine figure. Part 6 shows the difference between the two proteins, helix by helix. The secondary structure data, in SS3 format, is included to indicate significant changes in the secondary structure. Wheel figures of the helixes of both the original and designed proteins are embedded to illustrate the change in solubility and hydrophobic moment[23].

(B) Viewing 'Library Design' result

(i) Download the attached files. The library design results should contain two files, namely "*.out.txt" and "*.out2.txt". The '*' is a wildcard.

(ii) Open the file "*.out.txt" using a text file editing software program such as Notepad++. The file contains sequences in in multiple line format that is suitable for direct printing.

(iii) Open the file "*.out2.txt" with a text file editing software program such as Notepad++. This file is for the subsequent DNA synthesis, and is convenient for copy/paste operations. This file contain sequences in one-line mode, which can be easily read using a PC. In the files, all variants of each helix are listed in different lines. Open the file in Notepad++, with word wrap unchecked, and the user may easily observe the relationship between different peptide fragments in the designed library.

DNA design and synthesis ● TIMING 2 days – 3 weeks

14 | This step is for reverse translating the protein sequence to DNA sequence. It differs depending on whether a Simple Design (A) or Library Design (B) job is being submitted:

(A) DNA design for 'Simple Design'

Directly reverse translates the designed protein sequence to DNA sequence. This can be done with the assistance of JCat, the URL of which can be found on the 'Tools' page. The codon-usage bias of desired hosts and the subsequent cloning process should be considered here. Gene design services are available via many DNA synthesis company.

(B) DNA design for 'Library Design'

(i) Copy the divided fragments from the '*.out2.txt' file.

(ii) Reverse translate each fragment to DNA independently. Codon usage bias should be considered at this step.

(iii) Add overlapping sequences at the termini of the neighboring fragments.

(iv) Design primers for application of the DNA encoding whole length protein. The primers may be named as Pap1 and Pap2, respectively.

15 | This step is aimed at obtaining real DNA molecules and differs depending on whether a Simple Design (A) or Library Design (B) job is being submitted:

(A) DNA synthesis for 'Simple Design'

Perform a de novo DNA chemical synthesis by acquiring a commercial service. Usually, this process takes 2 – 7 days depending on the size of DNA and the selected company.

(B) DNA synthesis for 'Library Design'

(i) Chemically synthesize the designed DNA fragments separately.

(ii) Mix the synthesized DNA fragments together and perform a Non-standard PCR without Pap1 and Pap2. This is to join the fragments.

(iii) Perform PCR with Pap1 and Pap2 as the primers and the product of the last step as the template.

(iv) This entire process takes 2 – 4 weeks, depending on the protein size and selected company.

▲**CRITICAL STEP** Library synthesis is highly complicated and requires well trained technicians. Therefore, requesting professional service is strongly recommended. GenScript

(Nanjing, China) and Ginkgo Bioworks (Boston, USA, previous Gen9), who have established the method by collaborating with us are recommended. Therefore, their service may be requested as per user's convenience. The above stated 3-step assembly procedure is for normal-sized proteins. Assembly of large protein may require more steps.

Screening with Y2H ● TIMING 10 days

16 | Sub clone the library into Y2H vector to construct Y2H library.

17 | Perform yeast mating, and then screen the resulting cells with selection medium.

18 | Pick clones and verify them using colony PCR.

19 | Perform a re-mating for verification of screened transformants.

20 | Sequence the verified transformants to obtain the corresponding DNA sequences.

▲CRITICAL STEP Steps 16 – 20 can be skipped for 'Simple Design'. Y2H is well established. Reference manuals are available elsewhere. However, Y2H is yet a highly technical procedure, especially at the library construction step. Therefore, a professional company who is experience in Y2H should be contacted for service. In our previous work, we performed all Y2H experiments using the services of NextInteraction (Berkeley, USA).

Protein expression ● TIMING 3 days

21 | Clone the obtained DNA into an expression vector, and then cultivate the resulting cells for protein expression.

▲CRITICAL STEP One is encouraged to try different methods for conducting protein expression experiments, as this procedure is still in its formative stage and only several hosts have been tried. In regard to QTY designed human GPCRs, we have tried *E. coli* hosts, and insect cells. Insect cells are recommended for use due to the absence of inclusion-body issues. Other host systems, such as yeast system, may also be effective as Y2H always performs well for designed proteins, which indicates that the expression is fine in yeast. As for QTY variants screened from Y2H, their genes often need to be redesigned. This may be performed as per step 14(A) and 15(A) with a target expression host cell in mind, such as the insect cell.

22 | The expression of the proteins must be determined, followed by their purification and

characterization. The characterizing technique should be selected based on features of the protein concerned. For example, ligand binding was detected during the evaluation the QTY designed Chemokine receptors in our previous research[6].

Section2：

Data examples of Protein^{QTY}/Library^{QTY} design

Example 1

Name: CXCR4(C-X-C chemokine receptor type 4)

Uniprot Number: P61073

Protein sequence: (It is in the FASTA format. Simply copy, paste and save in a text editor software can make a file suitable for uploading)

```
>CXCR4
MEGISIYTSDNYTEEMGSGDYDSMKEPCFREENANFNKIFLPTIYSIIFLTGIVGNGLVI
LVMGYQKKLRSMTDKYRLHLSVADLLFVITLPFWAVDAVANWYFGNFLCKAVHVIYTVNL
YSSVLILAFISLDRYLAIVHATNSQRPRKLLAEKVVYVGVWIPALLLTIPDFIFANVSEA
DDRYICDRFYPNDLWVVVFQFQHIMVGLILPGIVILSCYCIIISKLSHSKGHQKRKALKT
TVILILAFFACWLPYYIGISIDSFILLEIIKQGCEFENTVHKWISITEALAFFHCCLNPI
LYAFLGAKFKTSAQHALTSVSRGSSLKILSKGKRGGHSSVSTESESSSFHSS
```

Secondary structure data: (It is in index format)

```
39,63,H
78,99,H
111,130,H
155,174,H
196,216,H
242,261,H
283,302,H
```

Secondary structure data: (It is in SS3 format)

```
CCCCCCCCCCCCCCCCCCCCCCCCCCCCCCCCCCCCCCHHHHHHHHHHHHHHHHHHHHHH
HHHCCCCCCCCCCCCCCHHHHHHHHHHHHHHHHHHHHHHCCCCCCCCCCCHHHHHHHHHH
HHHHHHHHHHCCCCEEEEEECCCCCCCCCCCCCCHHHHHHHHHHHHHHHHHHHHEECCCC
CCCEEEEEECCCCCCHHHHHHHHHHHHHHHHHHHHHCCCCCCCCCCCCCCCCCCCCCCCC
CHHHHHHHHHHHHHHHHHHHHCCCCCCCECCCCCCCCCCCCCCHHHHHHHHHHHHHHHHHH
HHCCCCCCCCCCCCCCCCCCCCCCCCCCCCCCCCCCCCCCCCCCCCCCCCCC
```

Example 2

Name: CLDN3(Claudin-3)

Uniprot Number: O15551

Protein sequence: (It is in the FASTA format. Simply copy, paste and save in a text editor software can make a file suitable for uploading)

```
>CLDN3
MSMGLEITGTALAVLGWLGTIVCCALPMWRVSAFIGSNIITSQNIWEGLWMNCVVQSTGQ
```

MQCKVYDSLLALPQDLQAARALIVVAILLAAFGLLVALVGAQCTNCVQDDTAKAKITIVA
GVLFLLAALLTLVPVSWSANTIIRDFYNPVVPEAQKREMGAGLYVGWAAAALQLLGGALL
CCSCPPREKKYTATKVVYSAPRSTGPGASLGTGYDRKDYV

Secondary structure data: (It is in index format)

1,8,Cytoplasmic
9,29,Helical
30,80,Extracellular
81,101,Helical
102,115,Cytoplasmic
116,136,Helical
137,159,Extracellular
160,180,Helical
181,220,Cytoplasmic

Secondary structure data: (It is in SS3 format)

CCCCCCCCHHHHHHHHHHHHHHHHHHHHHCCCCCCCCCCCCCCCCCCCCCCCCCCCCCCC
CCCCCCCCCCCCCCCCCCCCHHHHHHHHHHHHHHHHHHHHHCCCCCCCCCCCCCCHHHHH
HHHHHHHHHHHHHHHHEEEECCCCCCCCCCCCCCCCCCCHHHHHHHHHHHHHHHHHHHHH
CCCEEEEEECCCCCCCCCCCCCCCCCCCCCCCCCCCCCCC

Example 3

Name CCR5

Uniprot Number: P51681

Specified regions for design (It is in index format)

30,150
160,180
181,220

Section 3:

Simple Design report with annotations

REPORT OF QTY/NTY DESIGN

1. Job information:

Job/Protein Name: P51681

User: taofei@sjtu.edu.cn

Designing code: QTY

Length of protein sequence: 352

[illegible] of modified TM regions: 7

This is the name input by users. Here I used the Uniprot number as the protein name. This can be anything you want. But, one thing need to be aware. If you use the same name and the same email for different submissions. Only the last job will be kept for one week. All the submissions will be deleted after one week.

Means QTY was used in this design, it can also be NTY.

Means the original protein

2. Comparison of general characteristics:

This is for estimating the overall hydrophobicity of a protein. Kyte-Doolittle scale is used as the hydrophobic index.

Type	pI	MW (kDa)	Hydrophobicity	Mutation rate (TM, %)	Mutation rate (%)
WT	9.20	40.5241	0.5466	/	/
MT	9.07	41.0524	-0.9218	55.76	26.14

3. Comparison of TM prediction:

Means the QTY designed protein.

Means the mutation rate of trans-membrane regions. It was calculated by dividing the number of changed AA residues by the total number of residues in transmembrane regions

It was calculated by dividing the number of changed AA residues by the length of the protein sequence

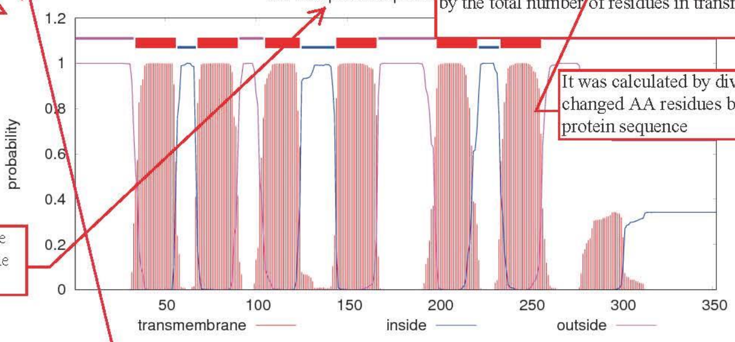

Was calculated using the wild type sequence as the input

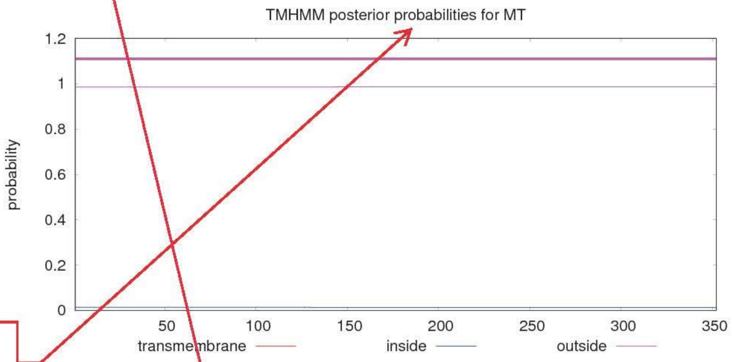

Was calculated using the variant sequence as the input.

This is done with the help of TMHMM 2.0. Every peak corresponds to a TM region. If there is no peak showing in the figure, that means all the TM regions have been removed from the protein.

4. Comparison of sequences:

Means identical

Means different

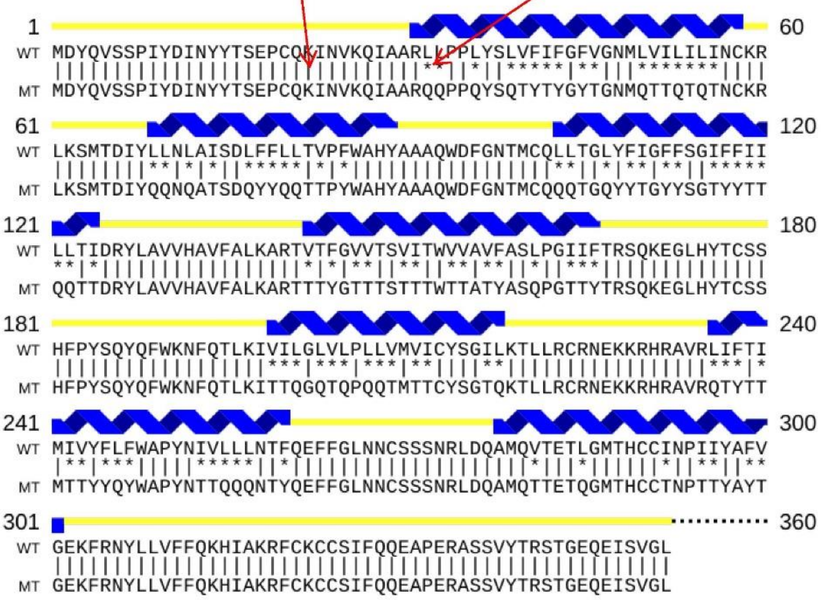

5. Comparison of Protter prediction:

This is completed by using the Protter sever. The TM region prediction and sub-cellular localization are all done by the server. It can clearly show that if there are still TM regions in a protein and if the protein is still in membrane.

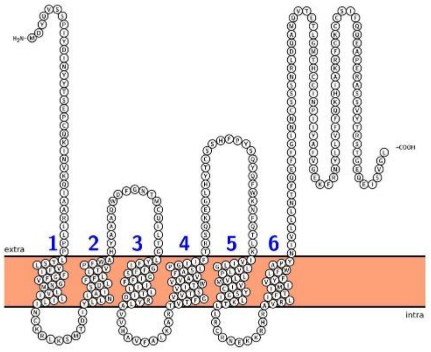

WT

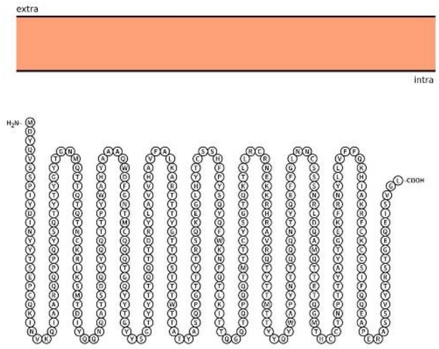

MT

6. Detailed comparison of TM regions:

Indicate the first trans-membrane helix

TM1

AA sequence comparison:

```
TM1-wt:  LLPPLYSLVFIFGFVGNMLVILILINCK
         **||*||*****|**|||*******|||
TM1-mt:  QQPPQYSQTYTYGYTGNMQTTQTQTNCK
```

The prediction was done with RaptorX

Alpha-helix prediction comparison:

```
TM1-wt:  HHHHHHHHHHHHHHHHHHHHHHHHHHHC
         |||||||||||||||||||||||||||*
TM1-mt:  HHHHHHHHHHHHHHHHHHHHHHHHHHHH
```

"H" means helix

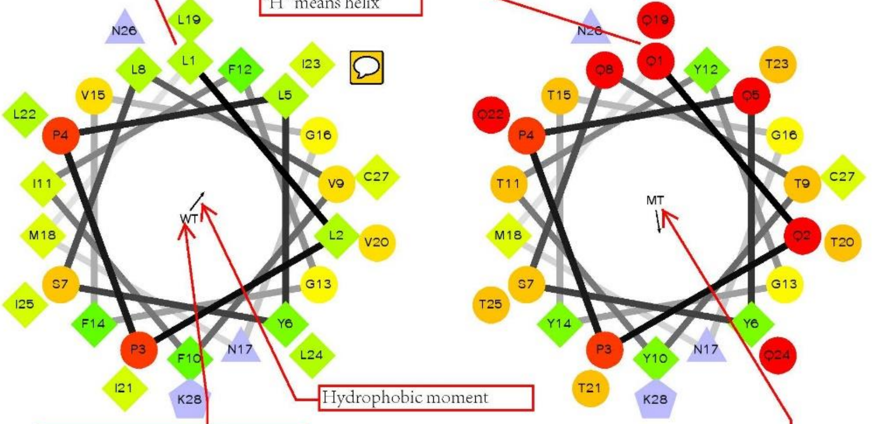

Hydrophobic moment

Wheel of the helix of wild type trans-membrane region

Wheel of the helix of QTY designed trans-membrane region

TM2

AA sequence comparison:

```
TM2-wt:  LNLAISDLFFLLTVPFWAHYA
         *|*|*||*****|*|*|||||
TM2-mt:  QNQATSDQYYQQTTPYWAHYA
```

Alpha-helix prediction comparison:

"C" means coil

```
TM2-wt:  HHHHHHHHHHHCCCHHHHHHH
         |||||||||||||||||||||
TM2-mt:  HHHHHHHHHHHCCCHHHHHHH
```

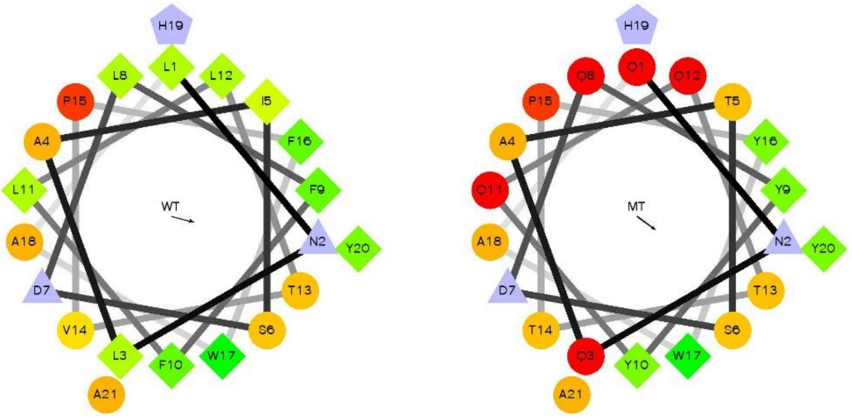

TM3

AA sequence comparison:

```
TM3-wt:  LTGLYFIGFFSGIFFIILLTID
         *||*|**|**||*******|*|
TM3-mt:  QTGQYYTGYYSGTYYTTQQTTD
```

Alpha-helix prediction comparison:

```
TM3-wt:  HHHHHHHHHHHHHHHHHHHHHH
         ||||||||||||||||||||||
TM3-mt:  HHHHHHHHHHHHHHHHHHHHHH
```

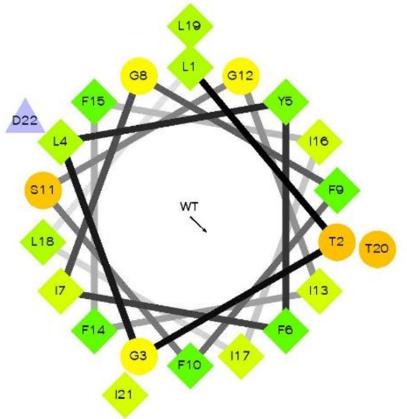

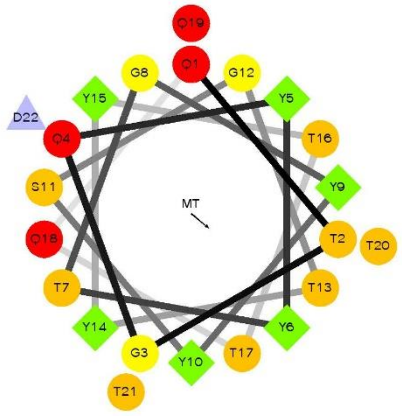

TM4

AA sequence comparison:

```
TM4-wt:  TFGVVTSVITWVVAVFASLPGIIFT
         |*|**||**||**|**||*||***|
TM4-mt:  TYGTTTSTTTWTTATYASQPGTTYT
```

Alpha-helix prediction comparison:

"E" means belta-sheet

```
TM4-wt:  HHHHHHHHHHHHHHHHHHCCCEEEE
         |||||||||||||||||*|||||||
TM4-mt:  HHHHHHHHHHHHHHHHHCCCCEEEE
```

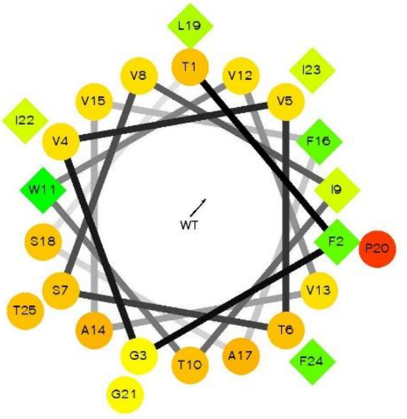

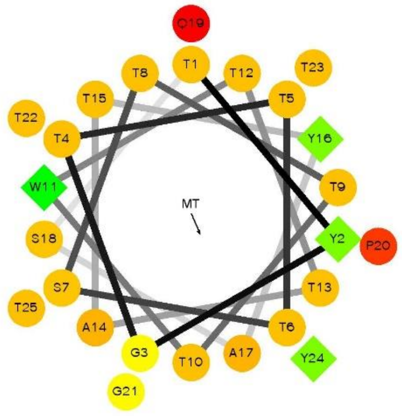

TM5

AA sequence comparison:

```
TM5-wt:  ILGLVLPLLVMVICYSGILK
         **|***|***|**||||**|
TM5-mt:  TQGQTQPQQTMTTCYSGTQK
```

Alpha-helix prediction comparison:

```
TM5-wt:  HHHHHHHHHHHHHHHHHHHH
         ||||||||||||||||||||
TM5-mt:  HHHHHHHHHHHHHHHHHHHH
```

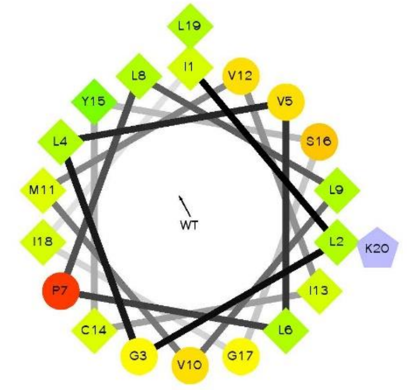

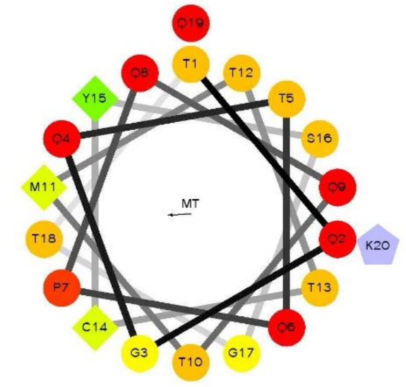

TM6

AA sequence comparison:

```
TM6-wt:  IFTIMIVYFLFWAPYNIVLLLNTFQ
         **|*|**|***|||||*****||*|
TM6-mt:  TYTTMTTYYQYWAPYNTTQQQNTYQ
```

Alpha-helix prediction comparison:

```
TM6-wt:  HHHHHHHHHHHCCHHHHHHHHHHHH
         |||||||||||||||||||||||||
TM6-mt:  HHHHHHHHHHHCCHHHHHHHHHHHH
```

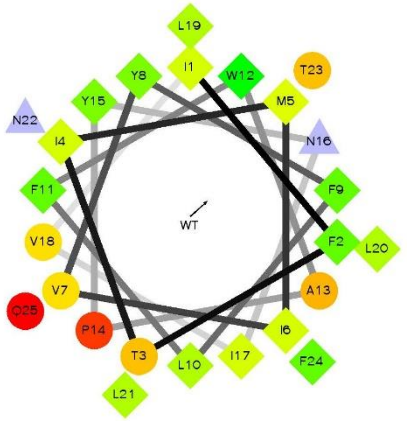

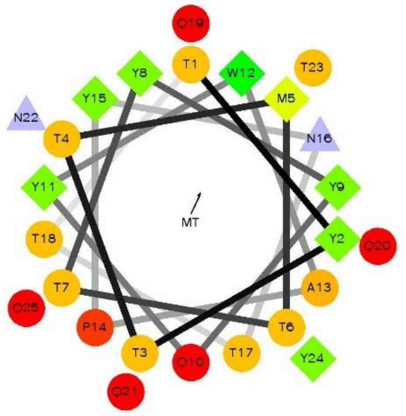

TM7

AA sequence comparison:

```
TM7-wt:  MQVTETLGMTHCCINPIIYAFVGE
         ||*|||*||||||*||**||**||
TM7-mt:  MQTTETQGMTHCCTNPTTYAYTGE
```

Alpha-helix prediction comparison:

```
TM7-wt:  HHHHHHHHHHHHHHHHHHHHHHCH
         |||||||||||****||||||*|*
TM7-mt:  HHHHHHHHHHHCCCCHHHHHHCCC
```

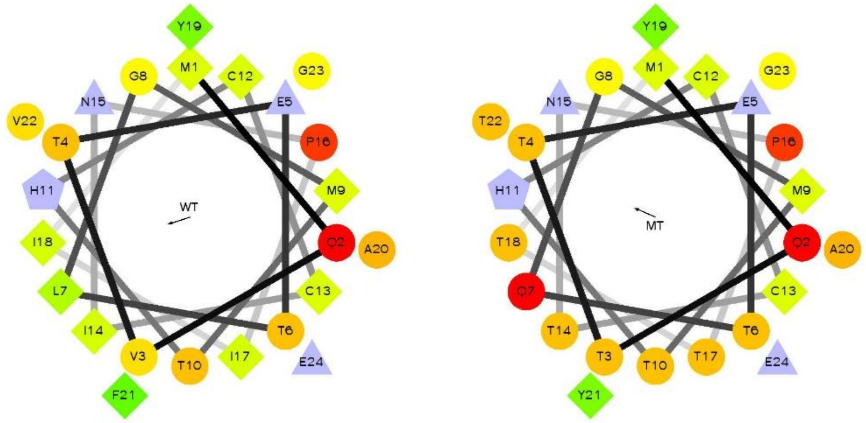

Section 4:

A library design result with annotations

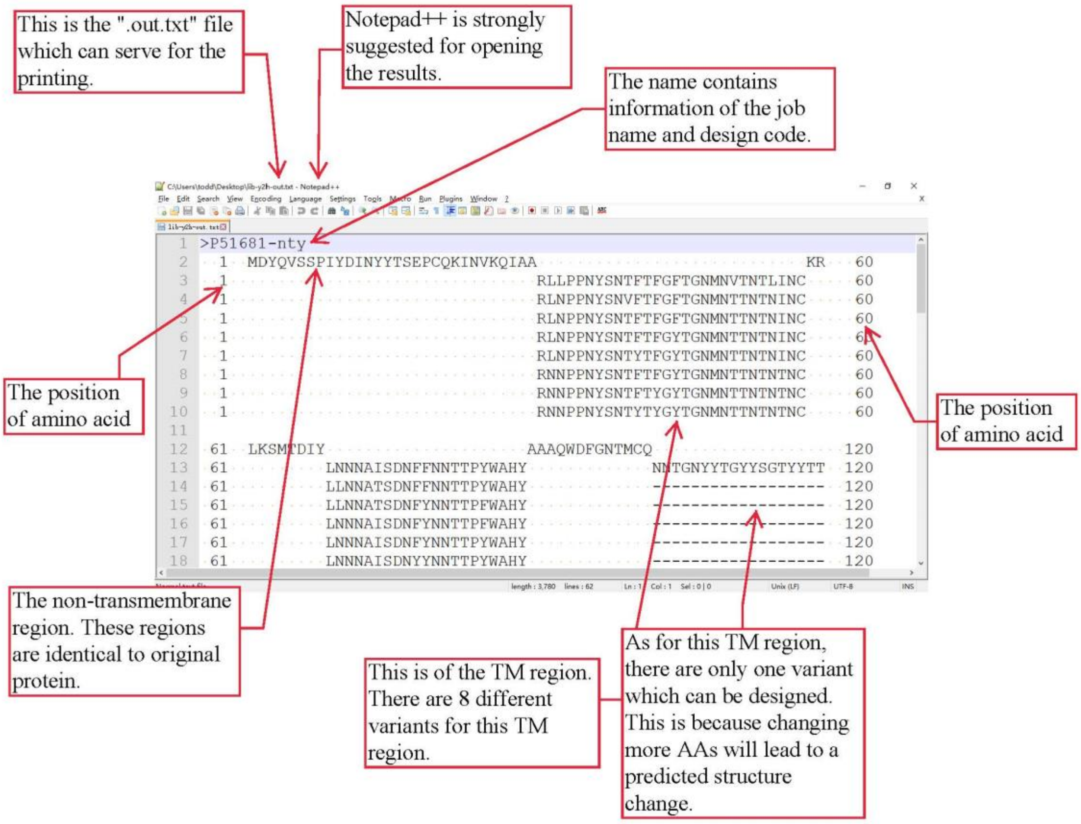

This is a full view of the whole result of the library design. It is easy to find that there are totally 7 TM regions and 8 non-TM regions.

```
>P51681-nty
  1  MDYQVSSPIYDINYYTSEPCQKINVKQIAA                            KR   60
  1                                RLLPPNYSNTFTFGFTGNMNVTNTLINC     60
  1                                RLNPPNYSNVFTFGFTGNMNTTNTNINC     60
  1                                RLNPPNYSNTFTFGFTGNMNTTNTNINC     60
  1                                RLNPPNYSNTFTFGYTGNMNTTNTNINC     60
  1                                RLNPPNYSNTYTFGYTGNMNTTNTNINC     60
  1                                RNNPPNYSNTFTFGYTGNMNTTNTNTNC     60
  1                                RNNPPNYSNTFTYGYTGNMNTTNTNTNC     60
  1                                RNNPPNYSNTYTYGYTGNMNTTNTNTNC     60

 61  LKSMTDIY                     AAAQWDFGNTMCQ                     120
 61          LNNNAISDNFFNNTTPYWAHY             NNTGNYYTGYYSGTYYTT  120
 61          LLNNATSDNFFNNTTPYWAHY             ------------------  120
 61          LLNNATSDNFYNNTTPFWAHY             ------------------  120
 61          LNNNAISDNFYNNTTPFWAHY             ------------------  120
 61          LNNNAISDNFYNNTTPYWAHY             ------------------  120
 61          LNNNAISDNYYNNTTPYWAHY             ------------------  120
 61          LNNNATSDNYYNNTTPYWAHY             ------------------  120
 61          NNNNATSDNYYNNTTPYWAHY             ------------------  120

121      DRYLAVVHAVFALKART                         TRSQKEGLHYTCSS  180
121  NNTT                 TTYGTTTSTTTWTTATYASNPGTTY                180
121  ----                 -------------------------                180
121  ----                 -------------------------                180
121  ----                 -------------------------                180
121  ----                 -------------------------                180
121  ----                 -------------------------                180
121  ----                 -------------------------                180
121  ----                 -------------------------                180

181  HFPYSQYQFWKNFQTLKI                    KTLLRCRNEKKRHRAVR       240
181                    VINGNVNPNNVMTTCYSGTL                 NTFTT  240
181                    VTNGNVNPNNTMVTCYSGIN                 NTFTT  240
181                    TINGNVNPNNTMVTCYSGTL                 NTFTT  240
181                    VTNGNVNPNNTMTICYSGTL                 NTFTT  240
181                    VTNGNVNPNNTMTTCYSGIN                 NTYTT  240
181                    TINGNTNPNNTMTTCYSGTL                 NTYTT  240
181                    TTNGNTNPNNTMVTCYSGTN                 NTYTT  240
181                    TTNGNTNPNNTMTTCYSGTN                 NTYTT  240

241                      QEFFGLNNCSSSNRLDQ                         300
241  MTTYFNFWAPYNTVNNLNTF                  AMQVTETNGMTHCCINPIIYAFV  300
241  MTTYYNFWAPYNTVNNLNTF                  AMQVTETNGMTHCCTNPIIYAFV  300
241  MTTYYNYWAPYNTVNNLNTF                  AMQVTETLGMTHCCTNPITYAFT  300
241  MTTYYNFWAPYNTVNNNNTF                  AMQVTETNGMTHCCTNPIIYAFT  300
241  MTTYYNFWAPYNTVNNNNTF                  AMQVTETNGMTHCCTNPTIYAFT  300
241  MTTYYNYWAPYNTVNNNNTF                  AMQVTETNGMTHCCTNPITYAYT  300
241  MTTYYNYWAPYNTTNNNNTF                  AMQTTETNGMTHCCTNPTTYAYV  300
241  MTTYYNYWAPYNTTNNNNTY                  AMQTTETNGMTHCCTNPTTYAYT  300

301   EKFRNYLLVFFQKHIAKRFCKCCSIFQQEAPERASSVYTRSTGEQEISVGL  352
301  G                                                     352
301  G                                                     352
301  G                                                     352
301  G                                                     352
301  G                                                     352
301  G                                                     352
301  G                                                     352
301  G                                                     352
```

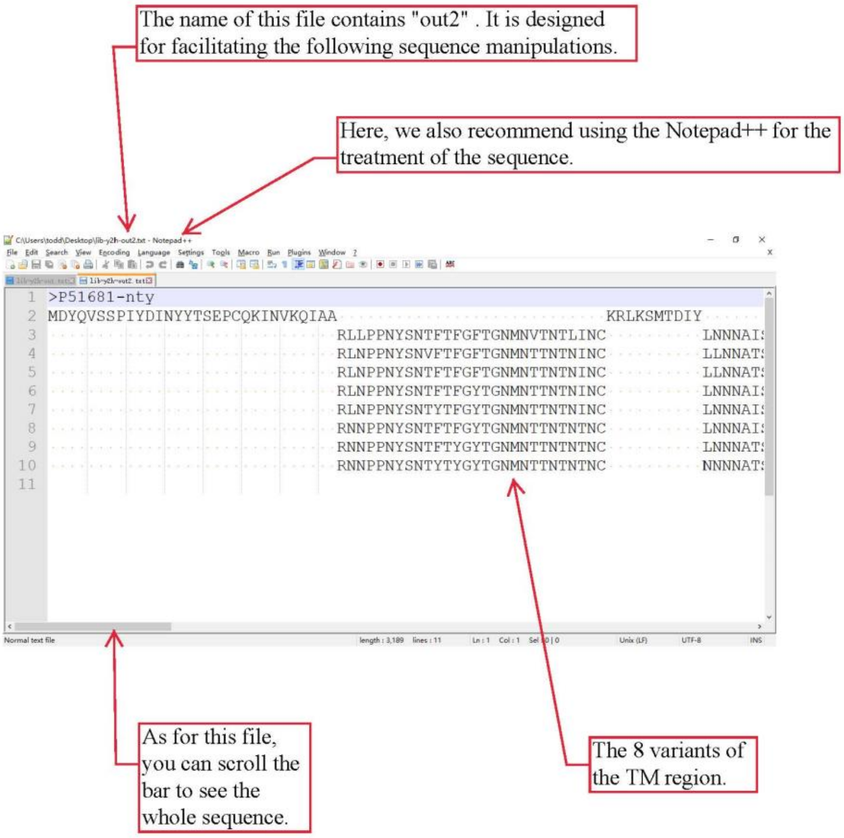
The name of this file contains "out2" . It is designed for facilitating the following sequence manipulations.
Here, we also recommend using the Notepad++ for the treatment of the sequence.
C:\Users\todd\Desktop\lib-y2h-out2.txt - Notepad++
File Edit Search View Encoding Language Settings Tools Macro Run Plugins Window ?
lib-y2h-out2.txt
>P51681-nty
MDYQVSSPIYDINYYTSEPCQKINVKQIAA KRLKSMTDIY
RLLPPNYSNTFTFGFTGNMNVTNTLINC LNNNAI
RLNPPNYSNVFTFGFTGNMNTTNTNINC LLNNAT
RLNPPNYSNTFTFGFTGNMNTTNTNINC LLNNAT
RLNPPNYSNTFTFGYTGNMNTTNTNINC LNNNAI
RLNPPNYSNTYTFGYTGNMNTTNTNINC LNNNAI
RNNPPNYSNTFTFGYTGNMNTTNTNTNC LNNNAI
RNNPPNYSNTFTYGYTGNMNTTNTNTNC LNNNAT
RNNPPNYSNTYTYGYTGNMNTTNTNTNC NNNNAT
As for this file, you can scroll the bar to see the whole sequence.
The 8 variants of the TM region.
